# Supplementary material for: Marylosides A-G, Norcycloartane Glycosides from Leaves of Cymbidium Great Flower ‘Marylaurencin’
Source: Molecules. 2019 Jul 9;24(13):2504. doi: 10.3390/molecules24132504 (PMC6651146; doi:10.3390/molecules24132504)

Compound 1

8-11(10-11)-3

exp5 hmqc

SAMPLE		DEC. & VT		ACQUISITION ARRAYS	
date	Jan 8 2010	dfrq	150.872	array	phase
solvent	CD3OD	dn	C13	arraydim	256
file /export/home/~		dpwr	45		
vnmr1/vnmrsys/data~		dof	-677.4	i	phase
/shou8/81110113_hm~		dm	nny	1	1
	qc.fid	dmm	ccp	2	2
ACQUISITION		dmf	13072		
sfrq	599.960	dseq	mpf7		
tn	H1	dres	1.0		
at	0.122	pwx	19.0		
np	1000	pwxlvl	57		
sw	4113.5	homo	n		
fb	not used	temp	25.0		
bs	4	PROCESSING			
ss	8	gf	0.056		
tpwr	60	gfs	not used		
pw	10.5	wtfile			
d1	0.900	proc	[illegible]		
tof	-1318.4	fn	2048		
nt	4	math	[illegible]		
ct	4				
alock	n	werr			
gain	4	wexp			
null	0.300	wbs			
j	150.0	wnt			
mbond	n	2D PROCESSING			
taumb	0	gf1	0.004		
satflg	nn	gfs1	not used		
satpwr	0	wtfile1			
satdly	0	proc1	lp		
satfrq	0	fn1	2048		
FLAGS					
il	y				
in	n				
dp	y				
hs	yy				
2D ACQUISITION					
sw1	31680.5				
ni	128				
phase	arrayed				
DISPLAY					
sp	176.6				
wp	2517.2				
vs	1000				
sc	10				
wc	270				
hzmm	9.33				
is	33.57				
rfl	2354.1				
rfp	1979.9				
th	3				
ins	100.000				
nm	ph				
2D DISPLAY					
sp1	1194.5				
wp1	15081.5				
sc2	0				
wc2	210				
rfl1	3017.2				
rfp1	0				

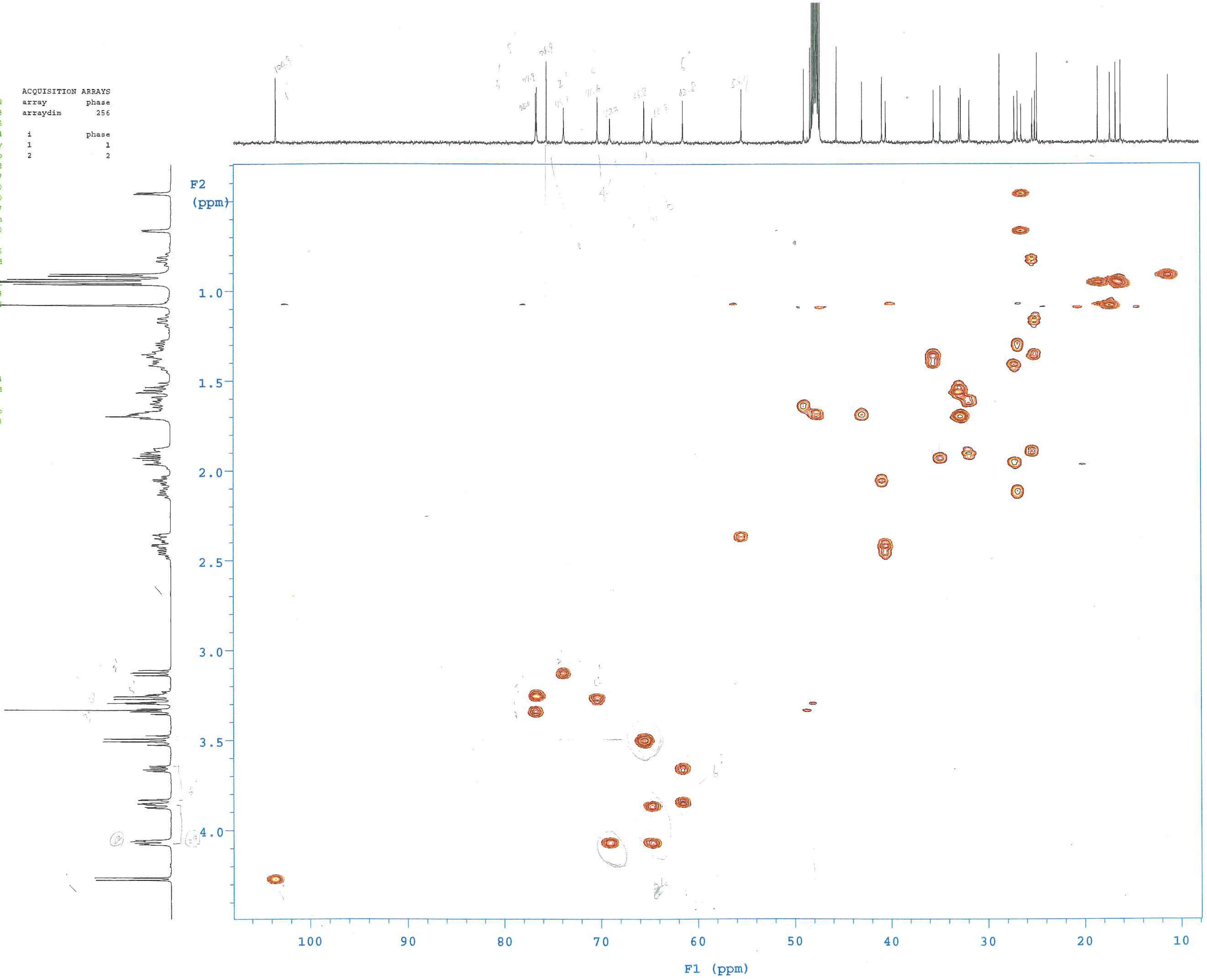

Compound 1

8-11(10-11)-3

exp3 gmqcosy

SAMPLE		DEC. & VT	
date	Jan 8 2010	dfrq	599.961
solvent	CD3OD	dn	H1
file	/export/home/~	dpwr	30
vnmr1/vnmrsys/data~		dof	0
/shou8/81110113_gm~		dm	nnn
	qcosy.fid	dmm	c
ACQUISITION		homo	n
sfrq	599.960	temp	25.0
tn	H1	GRADIENTS	
at	0.249	qlvl	2
np	2048	gzlvl1	10000
sw	4113.5	gt1	0.002500
fb	not used	grise	0.000010
bs	4	gstab	0
ss	2	taud2	0
tpwr	60	tau1	0
pw	10.5	PRESATURATION	
d1	1.500	satpwr	0
d2	0	satdly	0
tof	-1318.4	PROCESSING	
nt	1	sb	0.124
ct	1	sbs	not used
gain	60	proc	ft
FLAGS		fn	2048
dp	y		
hs	nn	werr	
2D ACQUISITION		wexp	
sw1	4115.2	wbs	
ni	256	wnt	wft
phase	1	2D PROCESSING	
DISPLAY		sb1	0.031
sp	176.6	sbs1	not used
wp	2517.2	proc1	ft
vs	2000	fn1	2048
sc	10		
wc	270		
rfl	2354.1		
rfp	1979.9		
th	7		
ins	100.000		
nm	av		
2D DISPLAY			
sp1	176.8		
wp1	2518.2		
sc2	0		
wc2	210		
rfl1	2354.1		
rfp1	1979.9		

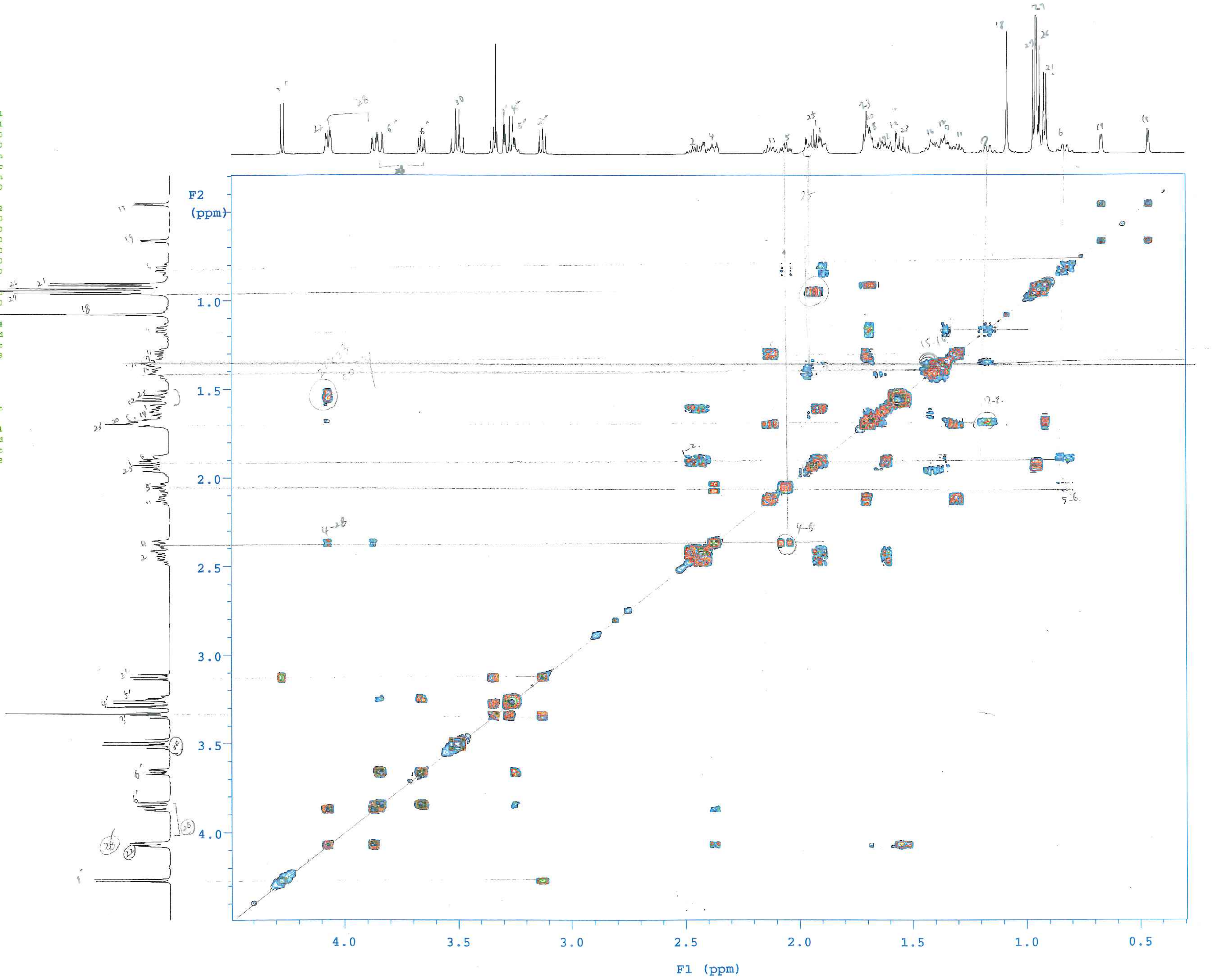

Compound 1

```
8-11(10-11)-3

exp6  gHMBC

      SAMPLE                  FLAGS
date    Jan  8 2010  hs                  n
solvent       CD3OD  sspul               n
sample    undefined  PFGflg              y
   ACQUISITION       hsglvl1         10256
sw           4113.5        SPECIAL
at            0.249  temp             25.0
np             2048  gain               60
fb         not used  spin                0
ss               32       GRADIENTS
d1            1.000  gzlvl1          10256
nt              512  gt1          0.001000
  2D ACQUISITION     gzlvl3           5128
sw1         37718.1  gt3          0.001000
ni              400  gstab        0.000500
phase             0    F2 PROCESSING
   TRANSMITTER       sb              0.124
tn               H1  sbs          not used
sfrq        599.960  fn               2048
tof         -1318.4    F1 PROCESSING
tpwr             60  sb1             0.005
pw           10.500  sbs1         not used
    DECOUPLER        fn1              2048
dn              C13         DISPLAY
dof          2339.8  sp              176.6
dm              nnn  wp             2517.2
dmm             ccc  sp1            1184.4
dmf           14815  wp1           15079.8
dpwr             46  rfl            2354.1
pwxlvl1          57  rfp            1979.9
pwx          19.000  rfl1           3018.8
       HMBC          rfp1                0
j1xh          140.0          PLOT
jnxh            8.0  wc              200.0
                     sc               10.0
                     wc2             210.0
                     sc2                 0
                     vs               1000
                     th                  7
                     nm     av
```

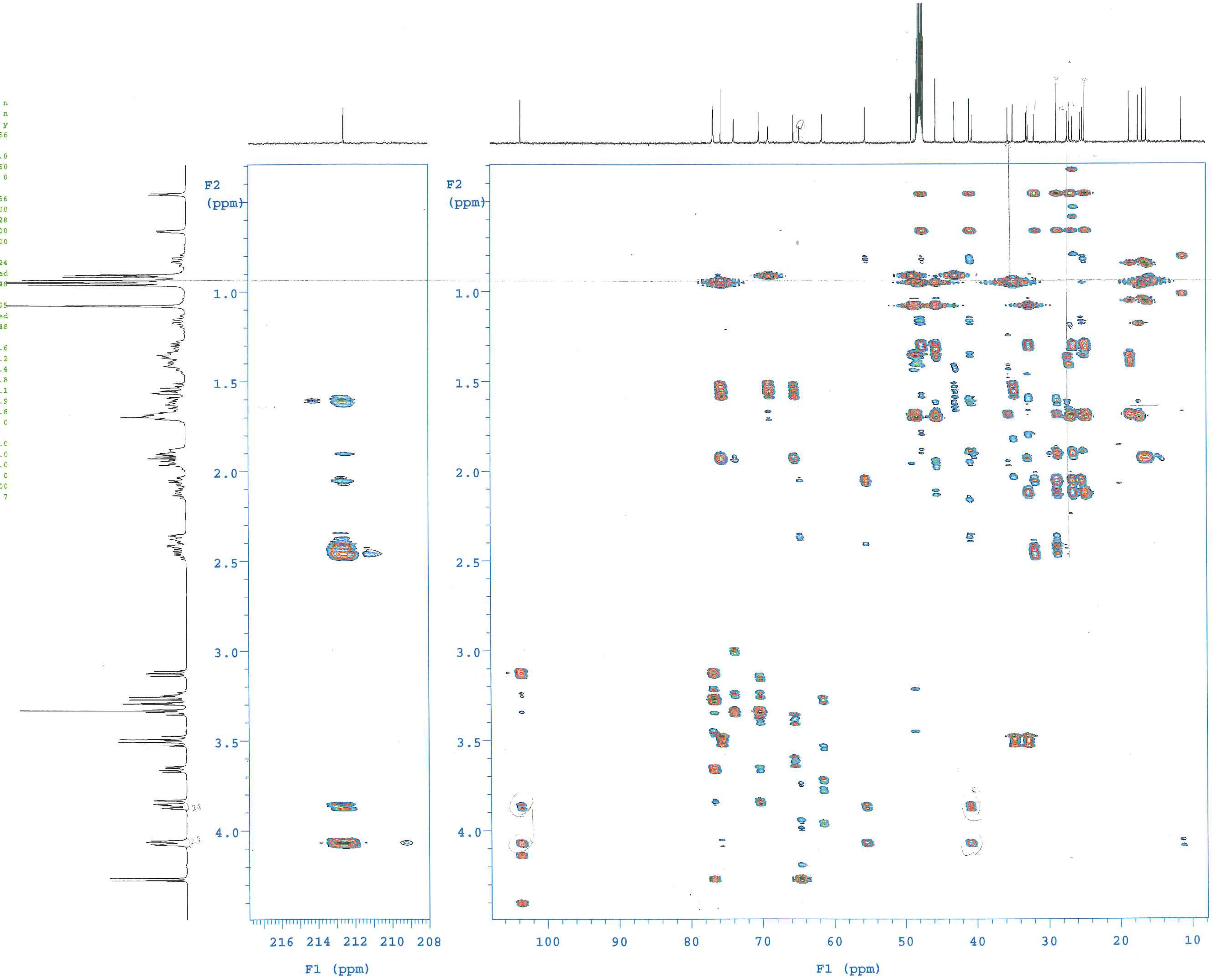

Compound 1

8-11(10-11)-3

exp4 noesy

SAMPLE		DEC. & VT		ACQUISITION ARRAYS	
date	Jan 8 2010	dfrq	599.961	array	phase
solvent	CD3OD	dn	H1	arraydim	256
file /export/home/~		dpwr	30		
vnmr1/vnmrsys/data~		dof	0	i	phase
/shou8/81110113_no~		dm	n	1	1
esy.fid		dmm	c	2	2
ACQUISITION		dmf	200		
sfrq	599.960	dseq			
tn	H1	dres	1.0		
at	0.249	homo	n		
np	2048	temp	25.0		
sw	4113.5	PROCESSING			
fb	not used	gf	0.115		
bs	4	gfs	not used		
ss	8	wtfile			
tpwr	60	proc	ft		
pw	10.5	fn	2048		
d1	1.500	math	f		
presat	0				
mix	0.800	werr			
tof	-1318.4	wexp			
nt	16	wbs			
ct	12	wnt	wft		
alock	n	2D PROCESSING			
gain	4	gf1	0.029		
FLAGS		gfs1	not used		
il	y	wtfile1			
in	n	proc1	lp		
dp	y	fn1	2048		
hs	yn				
sspul	n				
2D ACQUISITION					
sw1	4115.2				
ni	128				
phase	arrayed				
DISPLAY					
sp	176.6				
wp	2517.2				
vs	5155				
sc	10				
wc	270				
hzmm	9.33				
is	33.57				
rfl	2354.1				
rfp	1979.9				
th	1				
ins	100.000				
ai	ph				
2D DISPLAY					
sp1	176.8				
wp1	2518.2				
sc2	0				
wc2	210				
rfl1	2354.1				
rfp1	1979.9				

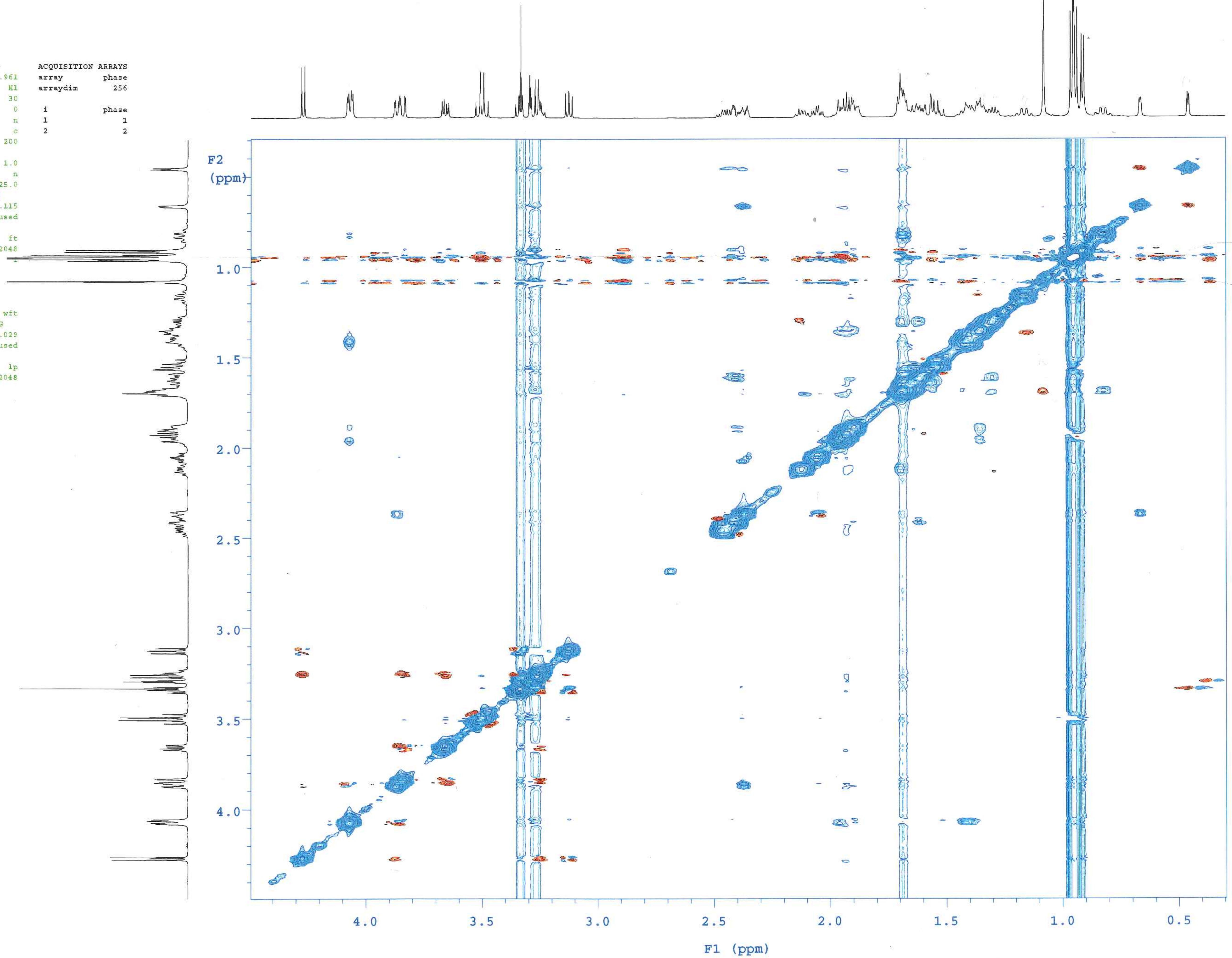

Compound 1

8-11(10-11)-3

exp8 roesy

SAMPLE		DEC. & VT		ACQUISITION ARRAYS	
date	Jan 8 2010	dfrq	599.961	array	phase
solvent	CD3OD	dn	H1	arraydim	256
file /export/home/-		dpwr	30		
vnmr1/vnmrsys/data-		dof	0	i	phase
/shou8/81110113_ro-		dm	nnn	1	1
	esy.fid	dmm	c	2	2
ACQUISITION		dmf	200		
sfrq	599.960	dseq			
tn	H1	dres	1.0		
at	0.249	homo	n		
np	2048	temp	25.0		
sw	4113.5	PROCESSING			
fb	not used	gf	0.115		
bs	4	gfs	not used		
ss	8	wtfile			
tpwr	60	proc	ft		
pw	3.5	fn	2048		
p1	10.5	math			
d1	1.500				
presat	0	werr			
tof	-1318.4	wexp			
ratio	5.7	wbs			
mix	0.600	wnt	wft		
nt	8	2D PROCESSING			
ct	8	gf1	0.029		
alock	n	gfs1	not used		
gain	4	wtfile1			
FLAGS		proc1	lp		
il	y	fn1	2048		
in	n				
dp	y				
hs	yn				
sspul	y				
rocomp	n				
2D ACQUISITION					
sw1	4115.2				
ni	128				
phase	arrayed				
DISPLAY					
sp	176.6				
wp	2517.2				
vs	5985				
sc	10				
wc	270				
hzmm	9.33				
is	33.57				
rfl	2354.1				
rfp	1979.9				
th	2				
ins	100.000				
nm	ph				
2D DISPLAY					
sp1	176.8				
wp1	2518.2				
sc2	0				
wc2	210				
rfl1	2354.1				
rfp1	1979.9				

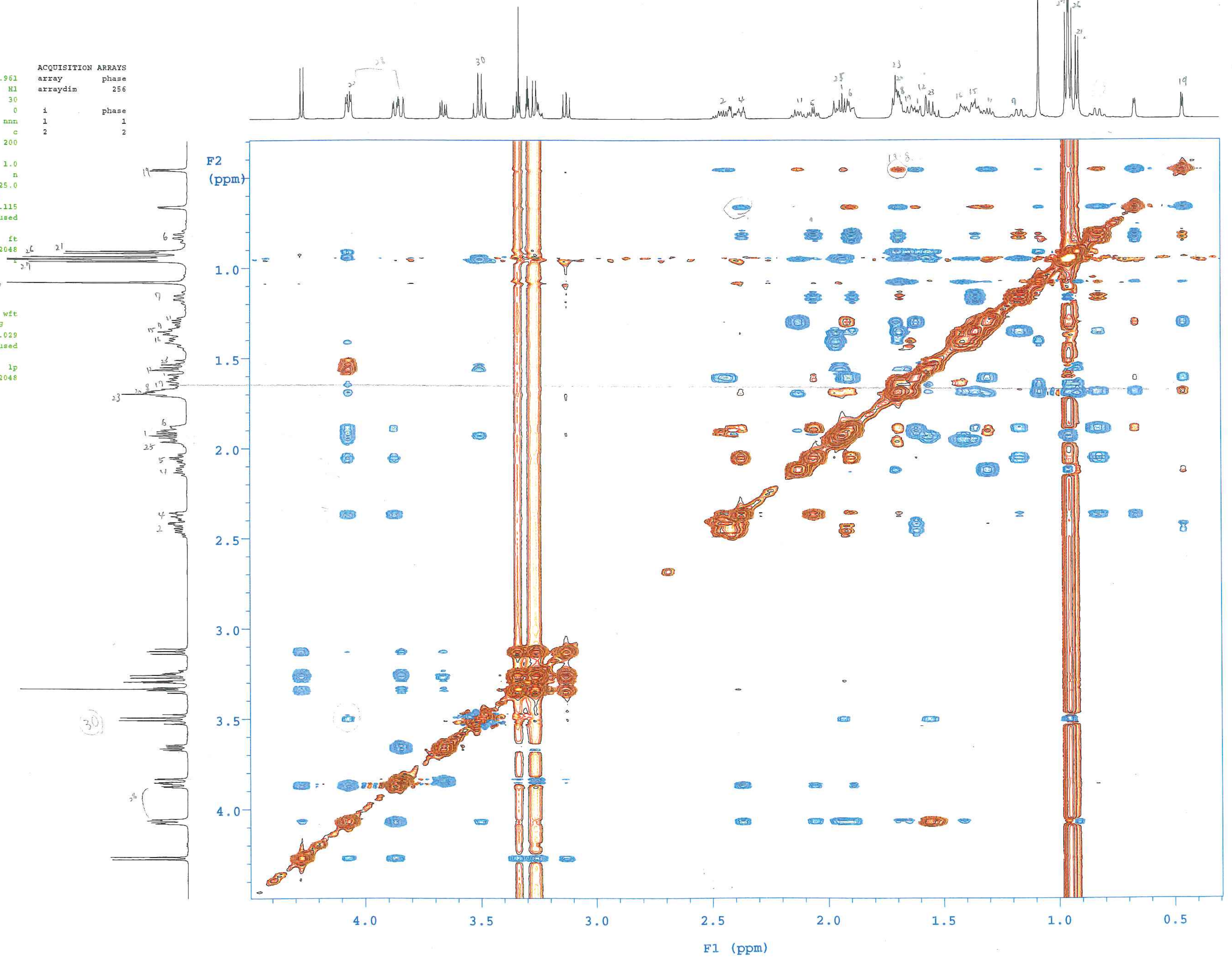

Compound 1a

```
Tani-48-54(33-40)

exp5  hmqc

      SAMPLE              DEC. & VT           ACQUISITION ARRAYS
date    Apr 27 2010  dfrq       150.872      array          phase
solvent   pyridine   dn             C13      arraydim         256
file /export/home/-  dpwr            45
vnmr1/vnmrsys/data-  dof         -677.4       i             phase
/shou11/tani485433-  dm             nny       1                 1
       40_hmqc.fid   dmm            ccp       2                 2
   ACQUISITION       dmf          13072
sfrq       599.957   dseq          mpf7
tn              H1   dres           1.0
at           0.080   pwx           19.0
np            1000   pwxlvl          57
sw          6250.0   homo             n
fb        not used   temp          25.0
bs               4      PROCESSING
ss               8   gf           0.037
tpwr            60   gfs       not used
pw            10.5   wtfile
d1           0.900   proc            ft
tof         -999.1   fn            2048
nt              16   math             f
ct              16
alock            n   werr
gain            42   wexp
null         0.300   wbs
j            150.0   wnt
mbond            n     2D PROCESSING
taumb            0   gf1          0.004
satflg          nn   gfs1      not used
satpwr           0   wtfile1
satdly           0   proc1           lp
satfrq           0   fn1           2048
      FLAGS
il               y
in               n
dp               y
hs              yy
  2D ACQUISITION
sw1        31680.4
ni             128
phase      arrayed
     DISPLAY
sp           115.3
wp          3775.7
vs            1651
sc              10
wc             270
hzmm         14.00
is           33.57
rfl         5629.5
rfp         5231.6
th               4
ins        100.000
nm    ph
    2D DISPLAY
sp1         1194.5
wp1        10838.8
sc2              0
wc2            210
rfl1        3017.2
rfp1             0
```

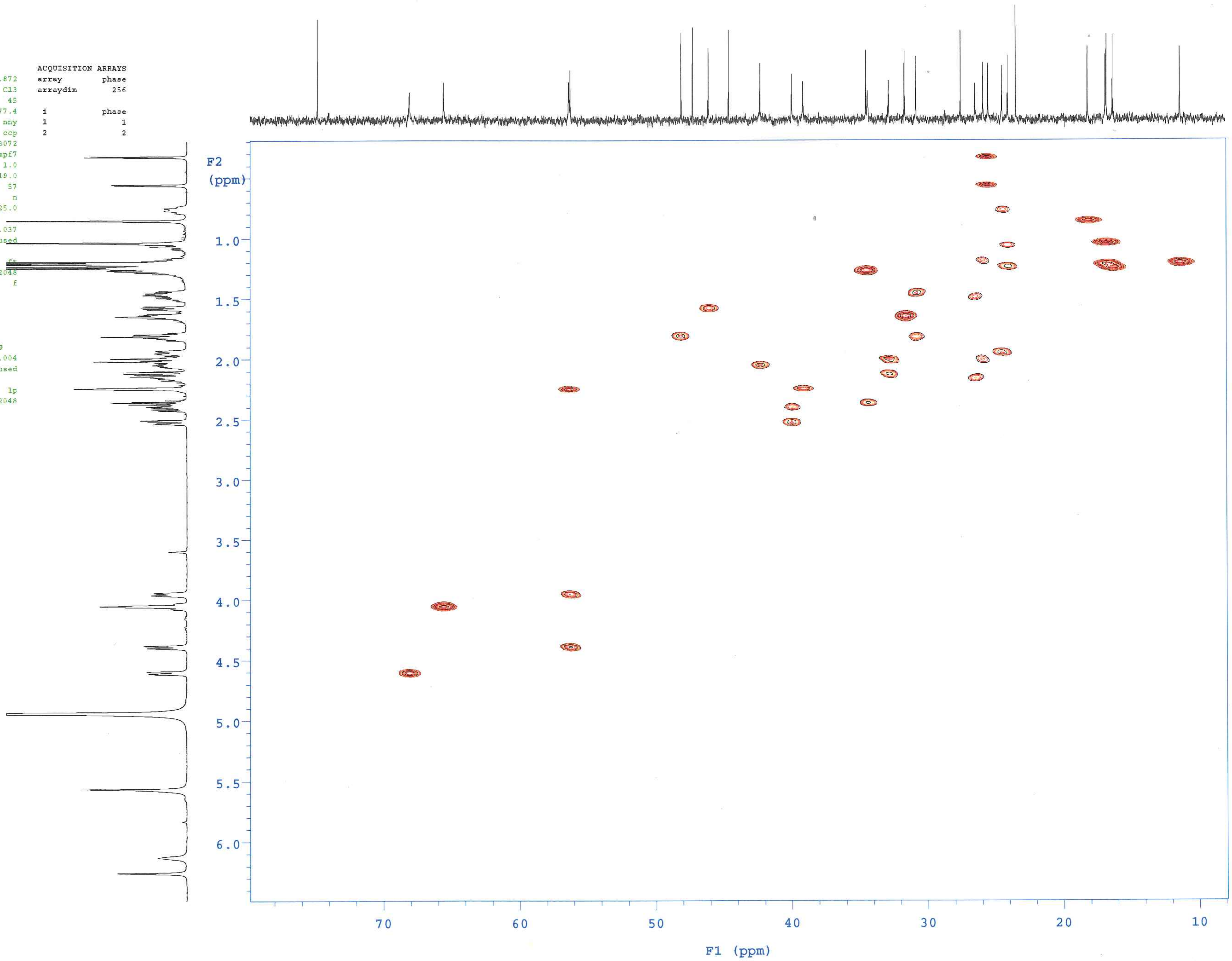

Compound 1a

Tani-48-54(33-40)

exp3 gmqcosy

```
     SAMPLE              DEC. & VT
date    Apr 27 2010  dfrq        599.958
solvent    pyridine  dn               H1
file /export/home/~ dpwr              30
vnmr1/vnmrsys/data~ dof                0
/shoul1/tani485433~ dm               nnn
   40_gmqcosy.fid    dmm               c
   ACQUISITION       homo              n
sfrq        599.957  temp           25.0
tn               H1       GRADIENTS
at            0.164  qlvl              2
np             2050  gzlvl1        10000
sw           6250.0  gt1        0.002500
fb         not used  grise      0.000010
bs                4  gstab             0
ss                2  taud2             0
tpwr             60  tau1              0
pw             10.5     PRESATURATION
d1            1.500  satpwr            0
d2                0  satdly            0
tof          -999.1       PROCESSING
nt                4  sb            0.082
ct                4  sbs        not used
gain             60  proc             ft
      FLAGS          fn             2048
dp                y
hs               nn  werr
  2D ACQUISITION     wexp
sw1          6245.1  wbs
ni              256  wnt             wft
phase             1    2D PROCESSING
     DISPLAY         sb1           0.020
sp            115.3  sbs1       not used
wp           3775.7  proc1            ft
vs             5155  fn1            2048
sc               10
wc              270
rfl          5629.5
rfp          5231.6
th                6
ins         100.000
ai    av
   2D DISPLAY
sp1           114.9
wp1          3778.8
sc2               0
wc2             210
rfl1         5629.5
rfp1         5231.6
```

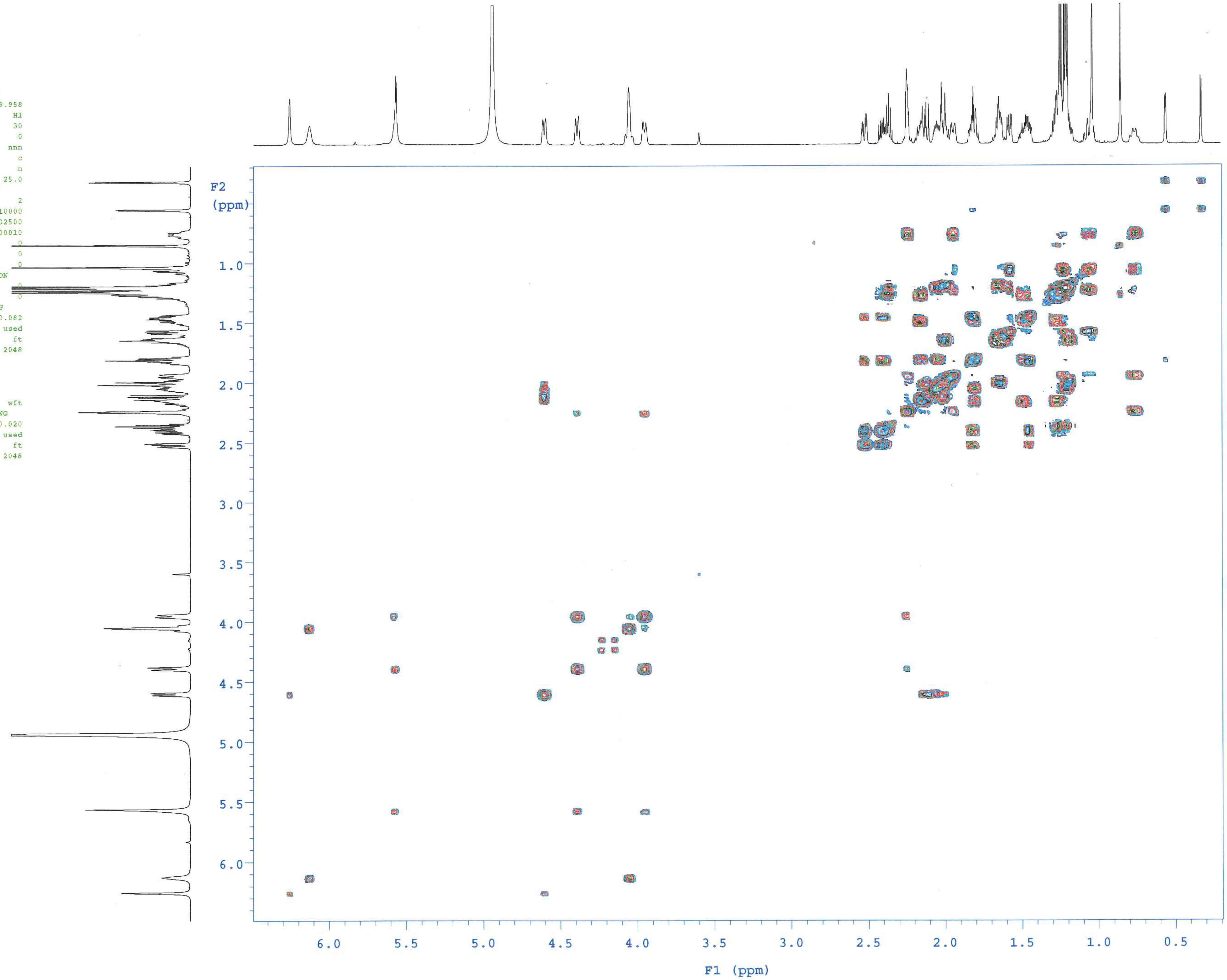

Compound 1a

Tani-48-54(33-40)

exp6 gHMBC

SAMPLE		FLAGS	
date	Apr 27 2010	hs	n
solvent	pyridine	sspul	n
sample	undefined	PFGflg	y
ACQUISITION		hsglvl	10256
sw	6250.0	SPECIAL	
at	0.164	temp	25.0
np	2048	gain	60
fb	not used	spin	0
ss	32	GRADIENTS	
d1	1.000	gzlvl1	10256
nt	512	gt1	0.001000
2D ACQUISITION		gzlvl3	5128
sw1	37718.1	gt3	0.001000
ni	400	gstab	0.000500
phase	0	F2 PROCESSING	
TRANSMITTER		sb	0.082
tn	H1	sbs	not used
sfrq	599.957	fn	2048
tof	-999.1	F1 PROCESSING	
tpwr	60	sb1	0.005
pw	10.500	sbs1	not used
DECOUPLER		fn1	2048
dn	C13	DISPLAY	
dof	2339.7	sp	115.3
dm	nnn	wp	3775.7
dmm	ccc	sp1	1184.3
dmf	14815	wp1	10839.8
dpwr	46	rfl	5629.5
pwxlvl	57	rfp	5231.6
pwx	19.000	rfl1	3018.8
HMBC		rfp1	0
j1xh	140.0	PLOT	
jnxh	8.0	wc	200.0
		sc	10.0
		wc2	210.0
		sc2	0
		vs	1651
		th	6
		nm	av

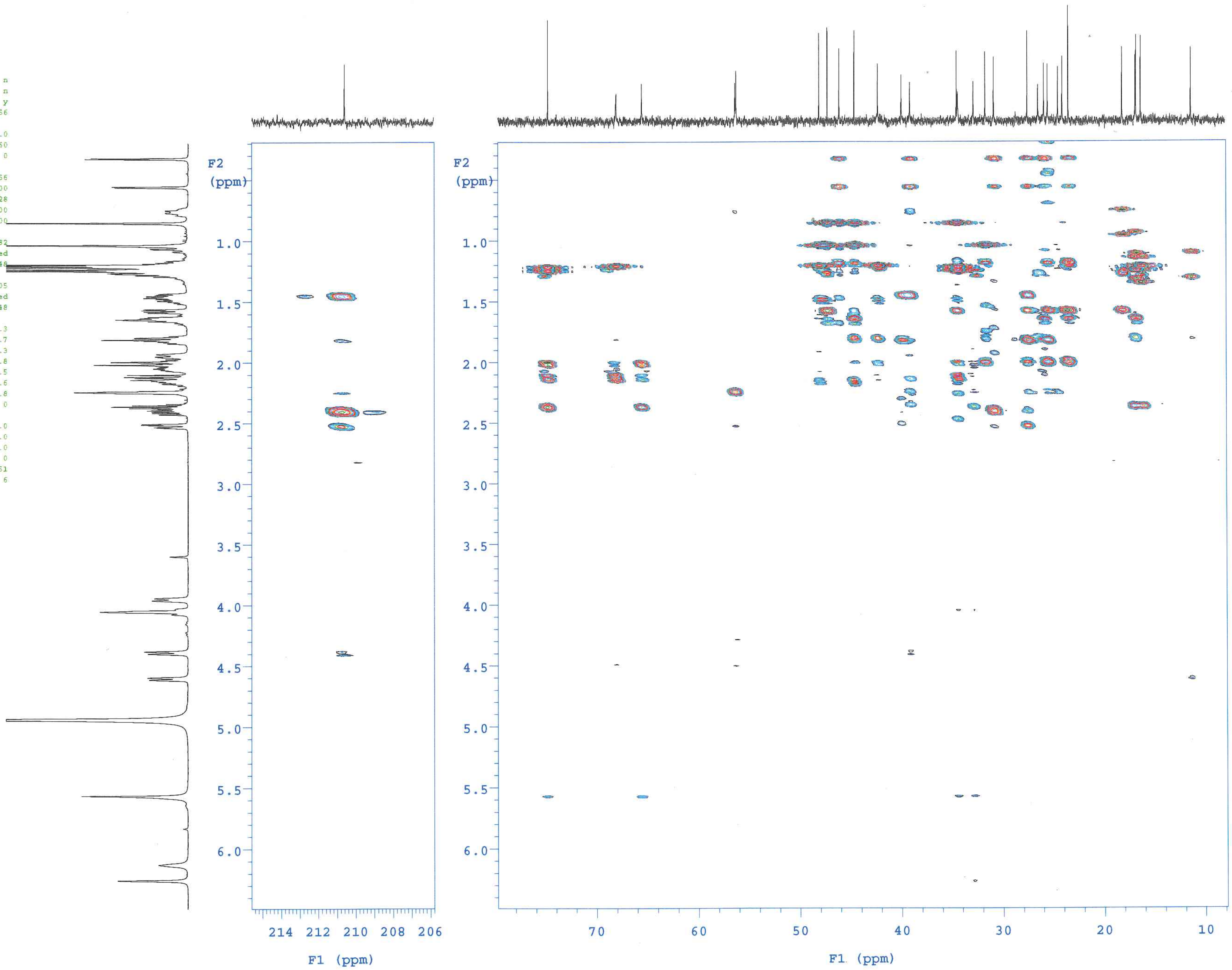

Compound 1a

```
Tani-48-54(33-40)

exp4  noesy

      SAMPLE              DEC. & VT          ACQUISITION ARRAYS
date   Apr 27 2010  dfrq        599.958     array        phase
solvent   pyridine  dn               H1     arraydim       256
file /export/home/~ dpwr             30
vnmr1/vnmrsys/data~ dof               0      i           phase
/shoul1/tani485433~ dm                n      1               1
      40_noesy.fid  dmm               c      2               2
   ACQUISITION      dmf             200
sfrq       599.957  dseq
tn              H1  dres            1.0
at           0.164  homo              n
np            2050  temp           25.0
sw          6250.0      PROCESSING
fb        not used  gf            0.076
bs               4  gfs        not used
ss               8  wtfile
tpwr            60  proc             ft
pw            10.5  fn             2048
d1           1.500  math              f
presat           0
mix          1.100  werr
tof         -999.1  wexp
nt              16  wbs
ct              16  wnt             wft
alock            n    2D PROCESSING
gain            26  gf1           0.019
     FLAGS          gfs1       not used
il               n  wtfile1
in               n  proc1            lp
dp               y  fn1            2048
hs              yn
sspul            n
  2D ACQUISITION
sw1         6245.1
ni             128
phase      arrayed
     DISPLAY
sp           115.3
wp          3775.7
vs           47756
sc              10
wc             270
hzmm         14.00
is           33.57
rfl         5629.5
rfp         5231.6
th               2
ins        100.000
ai    ph
    2D DISPLAY
sp1          114.9
wp1         3778.8
sc2              0
wc2            210
rfl1        5629.5
rfp1        5231.6
```

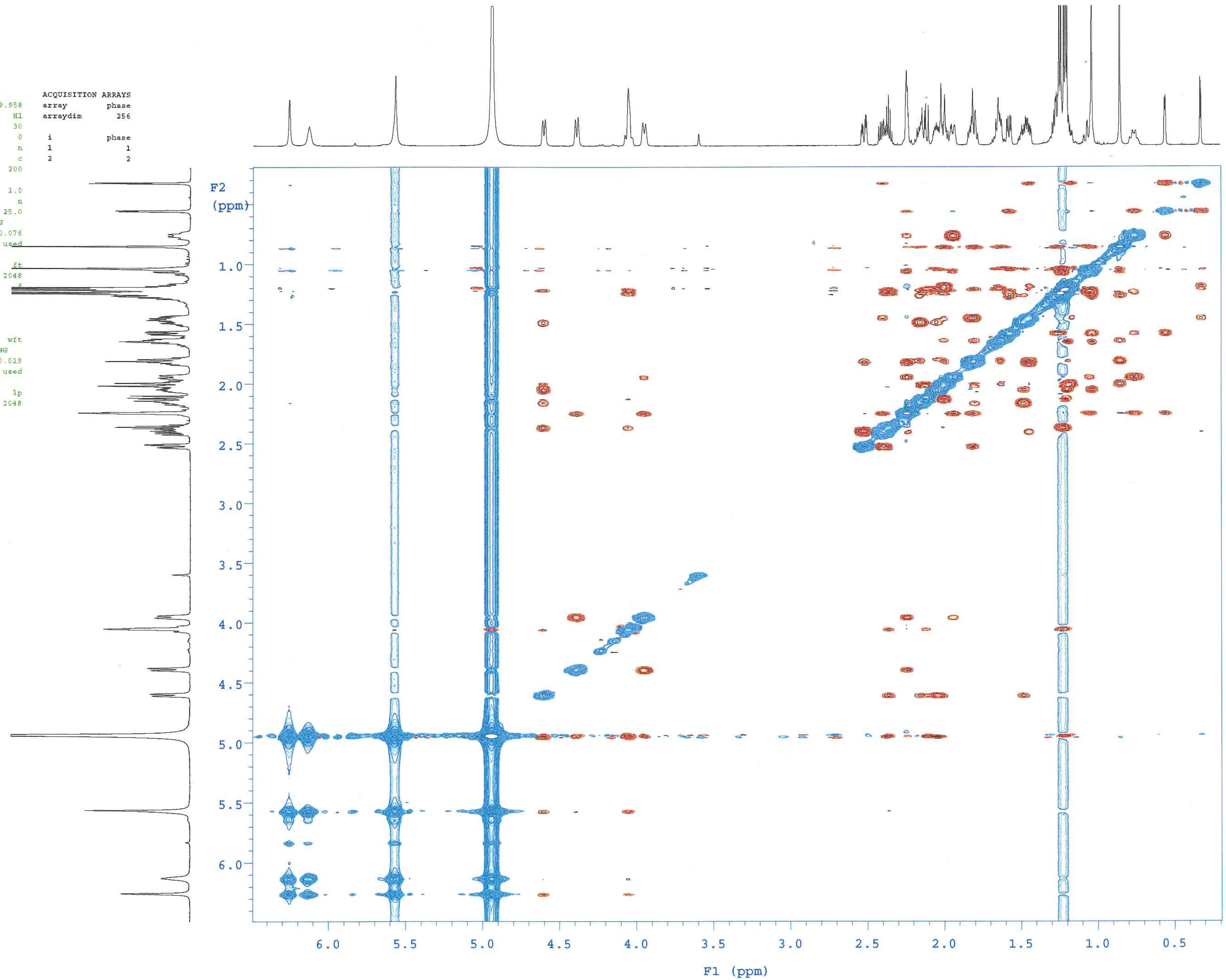

Compound 2

```
F26-30-8-9-10-38-56-60

exp5  hmqc

      SAMPLE              DEC. & VT          ACQUISITION ARRAYS
date    Jan 26 2011  dfrq         150.872    array          phase
solvent   pyridine   dn               C13    arraydim         256
file /export/home/-  dpwr              38
vnmrl/vnmrsys/data-  dof           -677.4     i             phase
/shou25/f263089103-  dm               nny     1                 1
    85660_hmqc.fid   dmm              ccp     2                 2
   ACQUISITION       dmf            13889
sfrq       599.957   dseq            mpf7
tn              H1   dres             1.0
at           0.081   pwx              8.5
np            1000   pwxlvl            56
sw          6172.4   homo               n
fb            3000   temp            25.0
bs               4       PROCESSING
ss               8   gf             0.037
tpwr            60   gfs         not used
pw            22.0   wtfile
d1           0.900   proc              ft
tof         -999.1   fn              2048
nt              16   math               f
ct               8
alock            n   werr
gain            36   wexp
null         0.300   wbs
j            150.0   wnt
mbond            n     2D PROCESSING
taumb            0   gf1            0.004
satflg          nn   gfs1        not used
satpwr           0   wtfile1
satdly           0   proc1             lp
satfrq           0   fn1             2048
      FLAGS
il               y
in               n
dp               y
hs              yy
  2D ACQUISITION
sw1        31680.4
ni             128
phase      arrayed
     DISPLAY
sp            55.3
wp          3297.2
vs            2457
sc              10
wc             270
hzmm         12.22
is           33.57
rfl         5592.2
rfp         5231.6
th               3
ins        100.000
ai    ph
    2D DISPLAY
sp1         1190.4
wp1        15376.1
sc2              0
wc2            210
rfl1        3017.2
rfp1             0
```

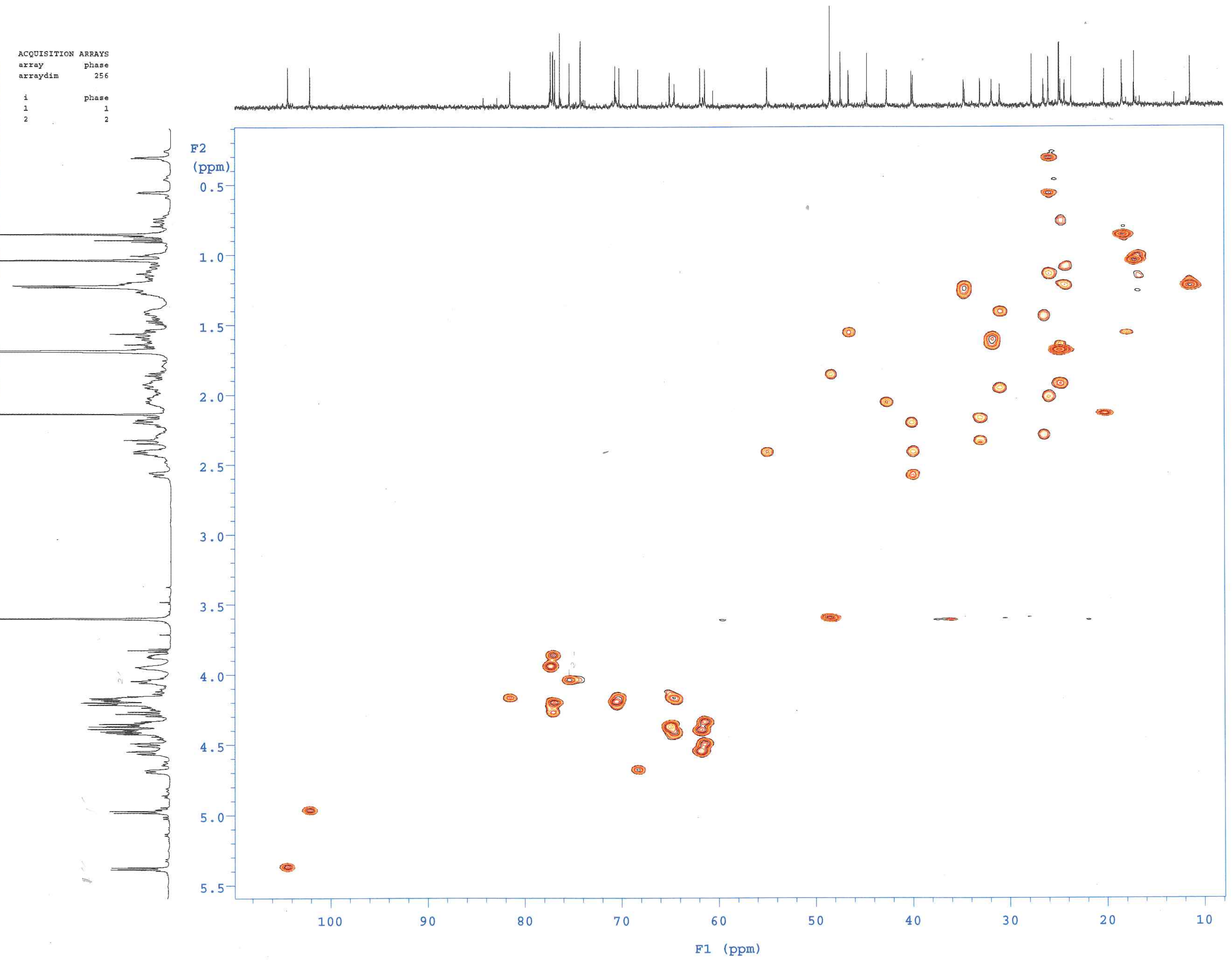

Compound 2

F26-30-8-9-10-38-56-60

exp3 gmqcosy

SAMPLE		DEC. & VT	
date	Jan 25 2011	dfrq	599.958
solvent	pyridine	dn	H1
file /export/home/~		dpwr	30
vnmr1/vnmrsys/data~		dof	0
/shou25/f263089103~		dm	nnn
85660_gmqcosy.fid		dmm	c
ACQUISITION		homo	n
sfrq	599.958	temp	25.0
tn	H1	GRADIENTS	
at	0.156	qlvl	2
np	2048	gzlvl1	10000
sw	6550.9	gt1	0.002500
fb	4000	grise	0.000010
bs	4	gstab	0
ss	2	taud2	0
tpwr	61	tau1	0
pw	10.9	PRESATURATION	
d1	1.500	satpwr	0
d2	0	satdly	0
tof	-869.1	PROCESSING	
nt	1	sb	0.078
ct	1	sbs	not used
gain	4	proc	ft
FLAGS		fn	2048
dp	y		
hs	nn	werr	
2D ACQUISITION		wexp	
sw1	6550.9	wbs	
ni	256	wnt	wft
phase	1	2D PROCESSING	
DISPLAY		sb1	0.019
sp	59.7	sbs1	not used
wp	3294.7	proc1	ft
vs	1148	fn1	2048
sc	10		
wc	270		
rfl	5651.7		
rfp	5231.6		
th	4		
ins	100.000		
ai	av		
2D DISPLAY			
sp1	59.7		
wp1	3294.7		
sc2	0		
wc2	210		
rfl1	5651.7		
rfp1	5231.6		

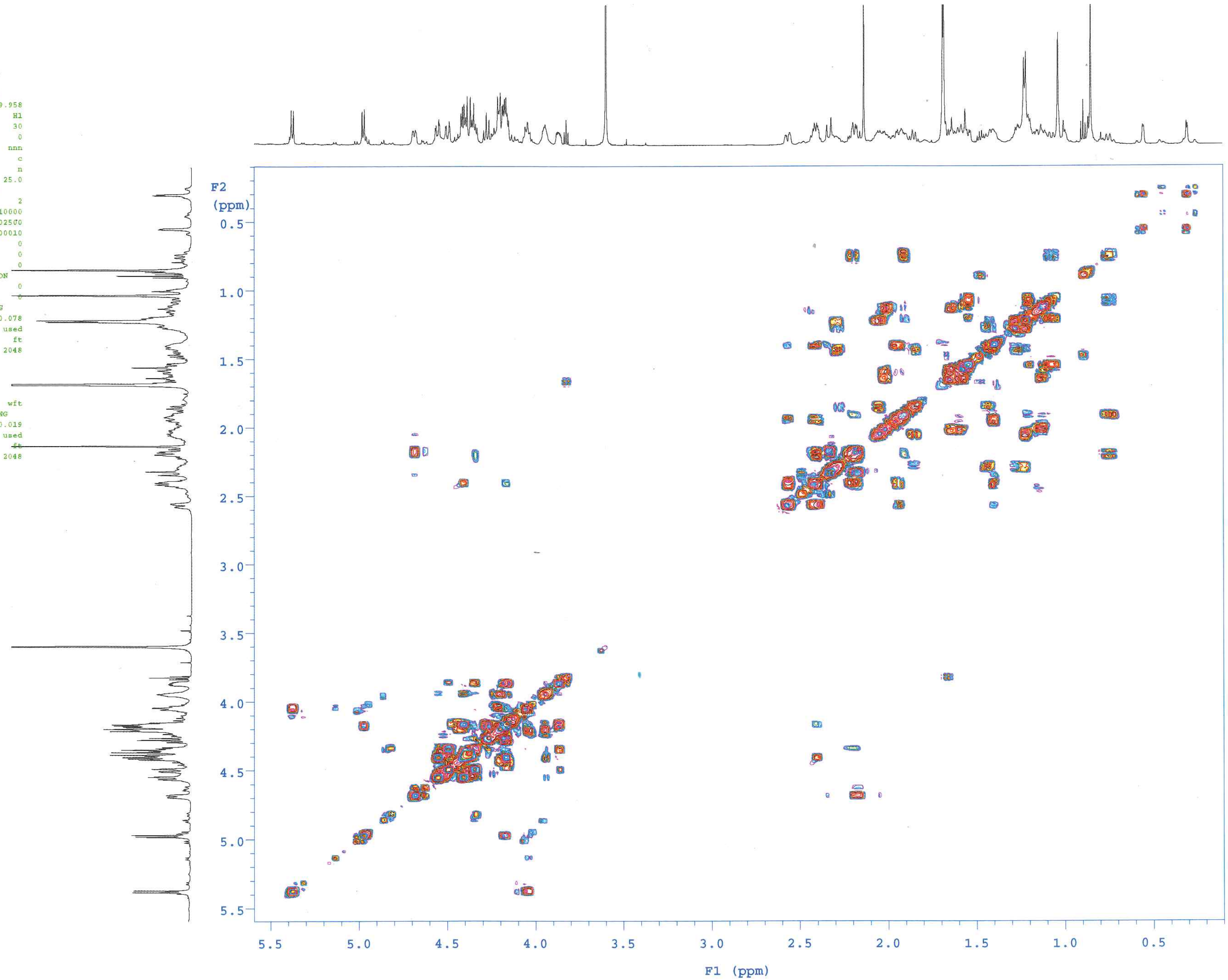

Compound 2

F26-30-8-9-10-38-56-60

exp6 gHMBC

SAMPLE		FLAGS	
date	Jan 25 2011	hs	n
solvent	pyridine	sspul	n
sample	undefined	PFGflg	y
ACQUISITION		hsglvl	10256
sw	6550.9	SPECIAL	
at	0.156	temp	25.0
np	2048	gain	60
fb	4000	spin	0
ss	32	GRADIENTS	
d1	1.000	gzlvl1	10256
nt	64	gt1	0.001000
2D ACQUISITION		gzlvl3	5128
sw1	37718.1	gt3	0.001000
ni	400	gstab	0.000500
phase	0	F2 PROCESSING	
TRANSMITTER		sb	0.078
tn	H1	sbs	not used
sfrq	599.958	fn	2048
tof	-869.1	F1 PROCESSING	
tpwr	61	sb1	0.005
pw	10.900	sbs1	not used
DECOUPLER		fn1	2048
dn	C13	DISPLAY	
dof	2339.7	sp	59.7
dm	nnn	wp	3294.7
dmm	ccc	sp1	1180.2
dmf	14815	wp1	31345.8
dpwr	46	rfl	5651.7
pwxlvl	57	rfp	5231.6
pwx	16.300	rfl1	3018.8
HMBC		rfp1	0
j1xh	140.0	PLOT	
jnxh	8.0	wc	270.0
		sc	10.0
		wc2	210.0
		sc2	0
		vs	2457
		th	6
		ai	av

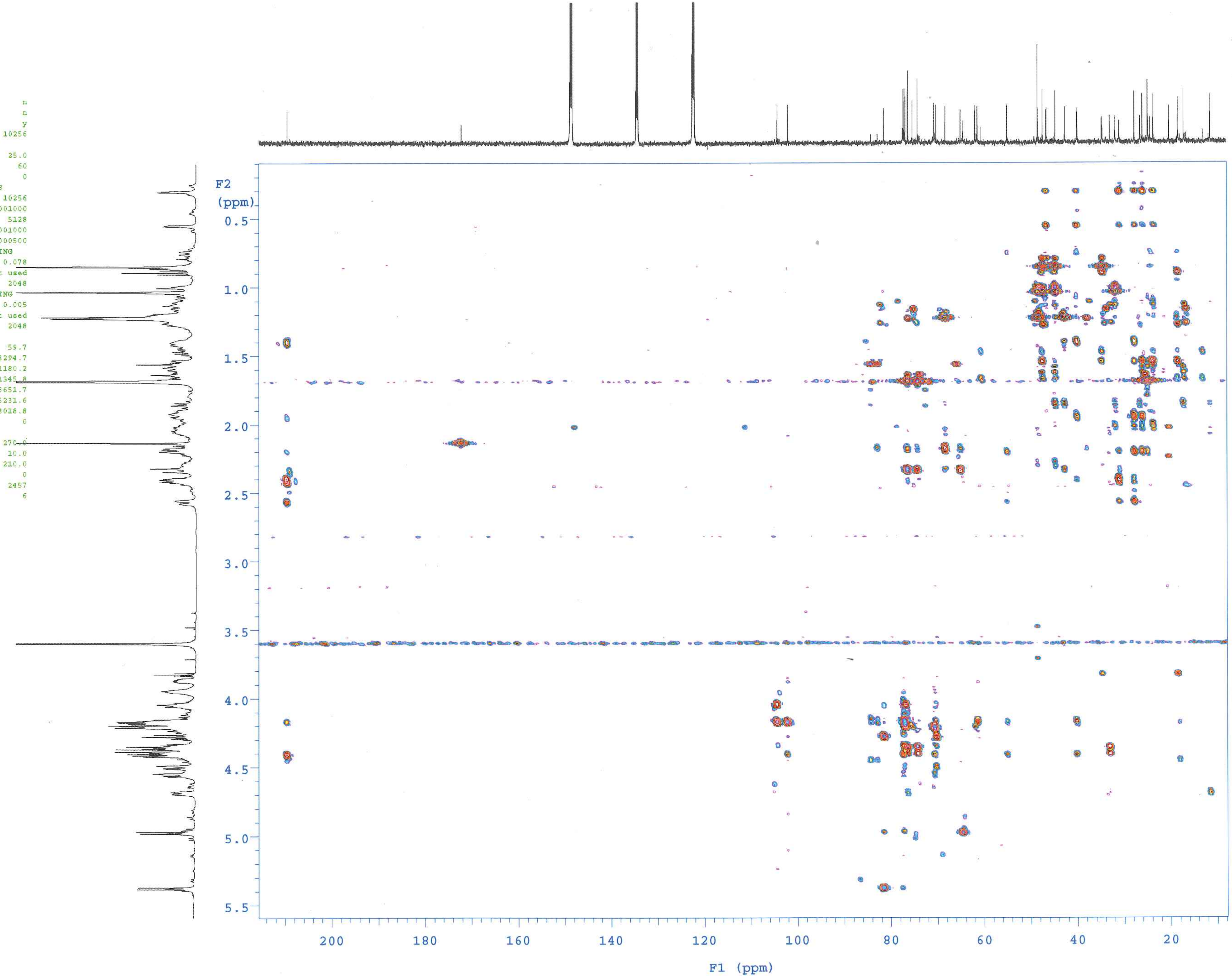

Compound 2

```
F26-30-8-9-10-38-56-60

exp4  roesy

     SAMPLE              DEC. & VT            ACQUISITION ARRAYS
date   Jan 25 2011  dfrq        599.958       array          phase
solvent   pyridine  dn               H1       arraydim         256
file /export/home/- dpwr             30
vnmrl/vnmrsys/data- dof               0        i             phase
/shou25/f263089103- dm              nnn        1                 1
   85660_roesy.fid  dmm               c        2                 2
   ACQUISITION      dmf             200
sfrq       599.958  dseq
tn              H1  dres            1.0
at           0.156  homo              n
np            2048  temp           25.0
sw          6550.9      PROCESSING
fb            4000  gf            0.072
bs               4  gfs        not used
ss               8  wtfile
tpwr            61  proc             ft
pw             3.6  fn             2048
p1            10.9  math              f
d1           1.500
presat           0  werr
tof         -869.1  wexp
ratio          3.0  wbs
mix          0.600  wnt             wft
nt              16    2D PROCESSING
ct               8  gf1           0.018
alock            n  gfs1       not used
gain             4  wtfile1
     FLAGS          proc1            lp
il               y  fn1            2048
in               n
dp               y
hs              yn
sspul            y
rocomp           n
  2D ACQUISITION
sw1         6550.9
ni             128
phase      arrayed
     DISPLAY
sp            59.7
wp          3294.7
vs             100
sc              10
wc             270
hzmm         12.22
is           33.57
rfl         5651.7
rfp         5231.6
th               2
ins        100.000
ai    ph
   2D DISPLAY
sp1           59.7
wp1         3294.7
sc2              0
wc2            210
rfl1        5651.7
rfp1        5231.6
```

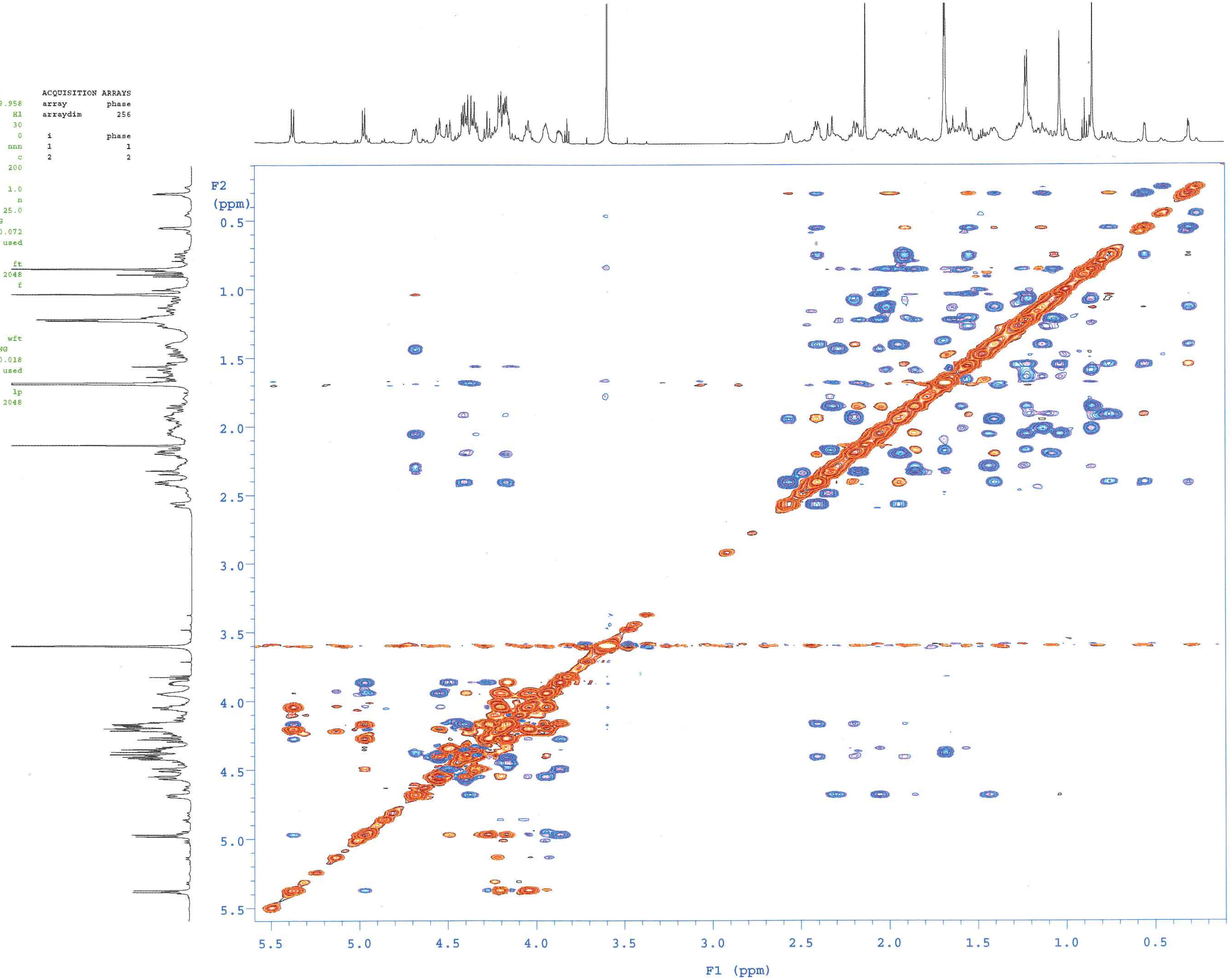

Compound 3 HMQC

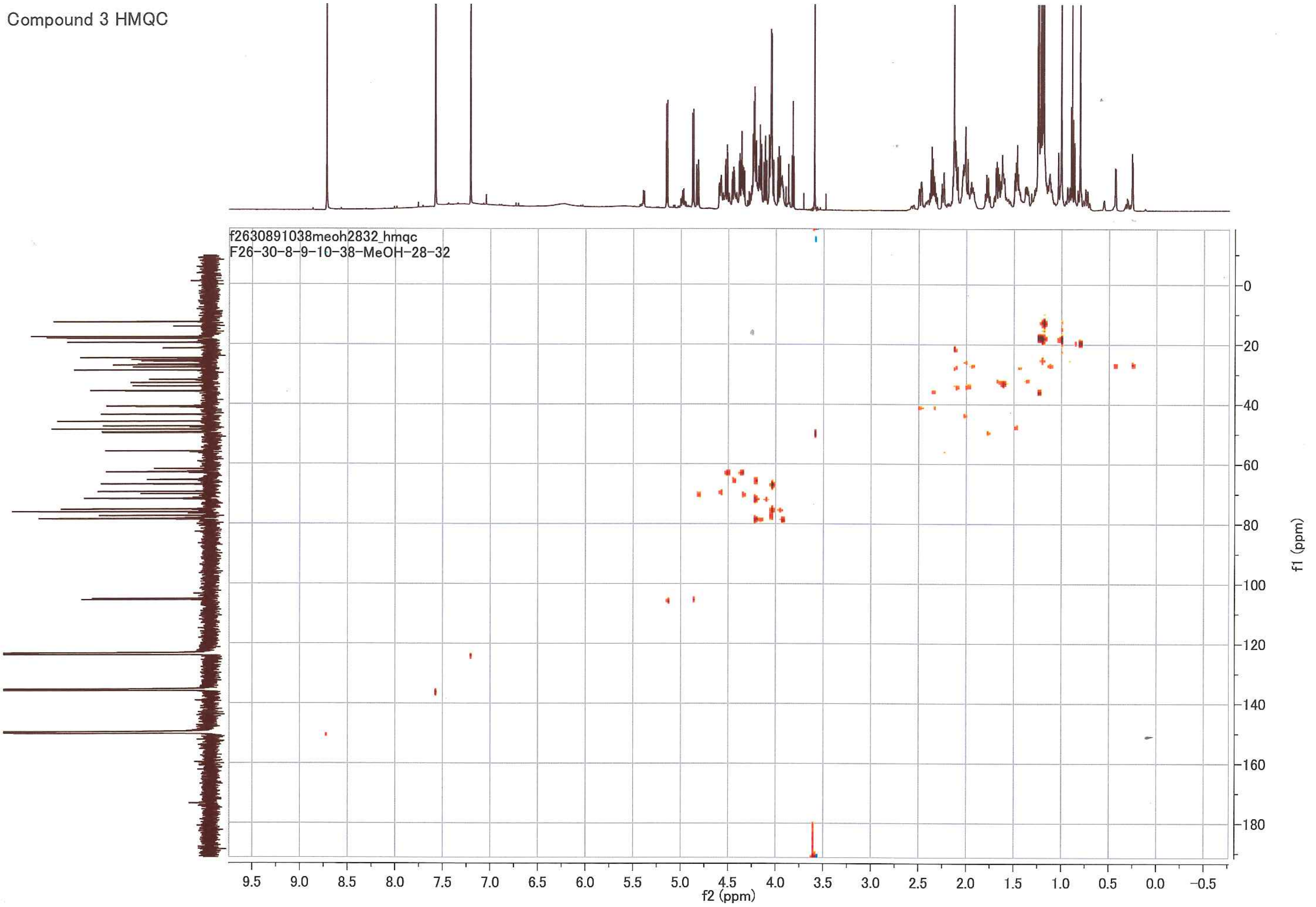

Compound 3 COSY

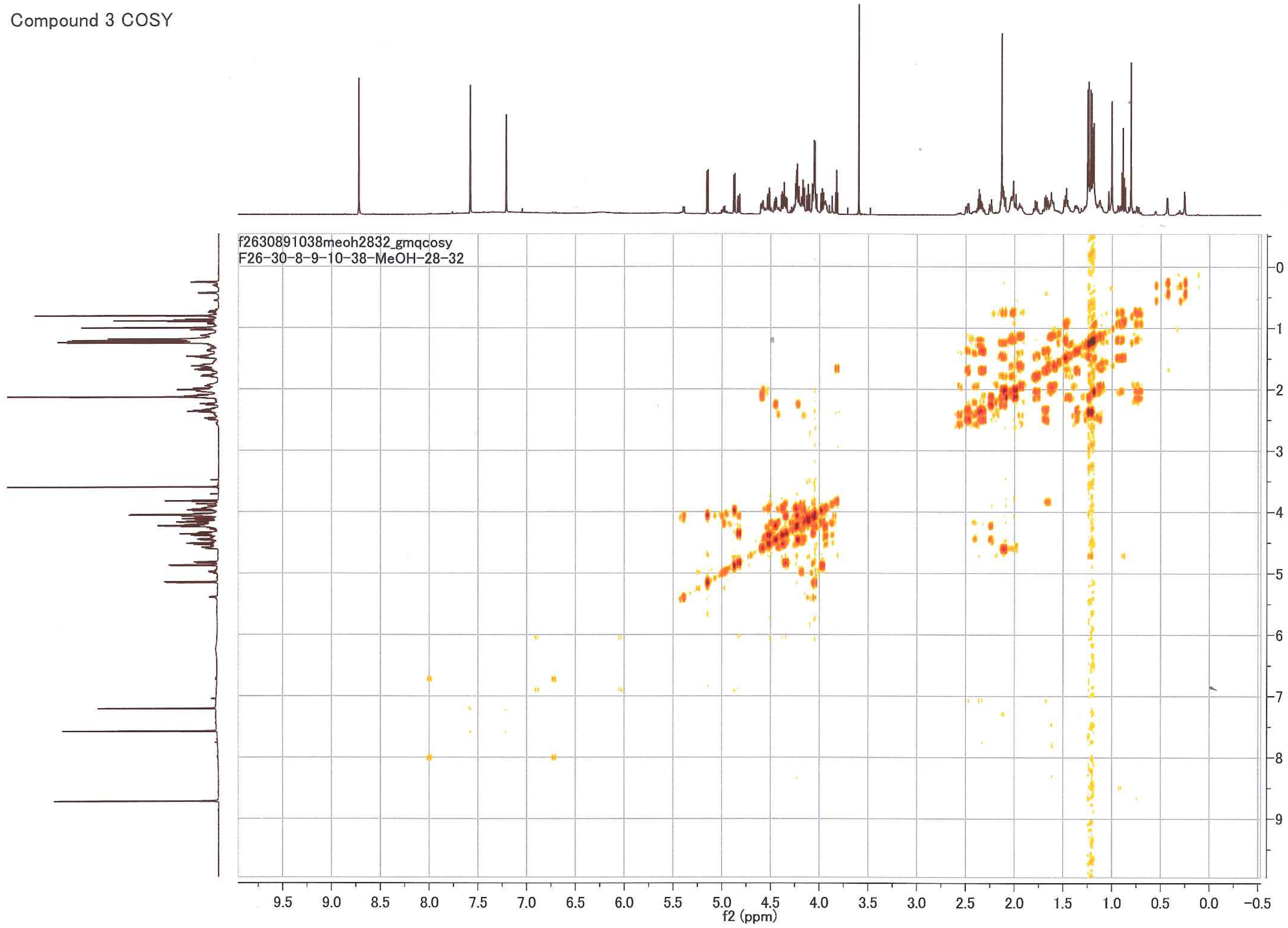

Compound 3 HMBC

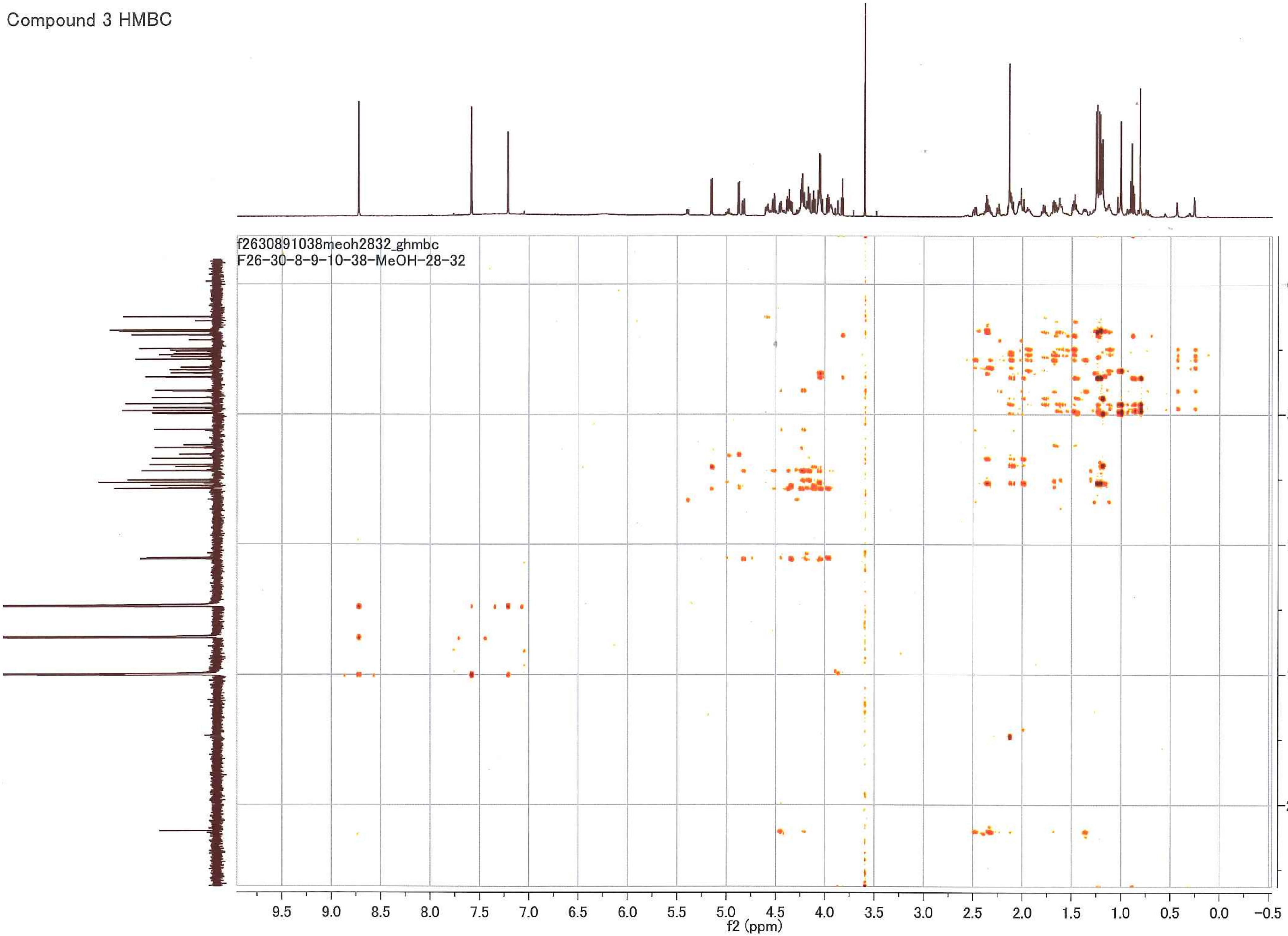

Compound 3 ROESY

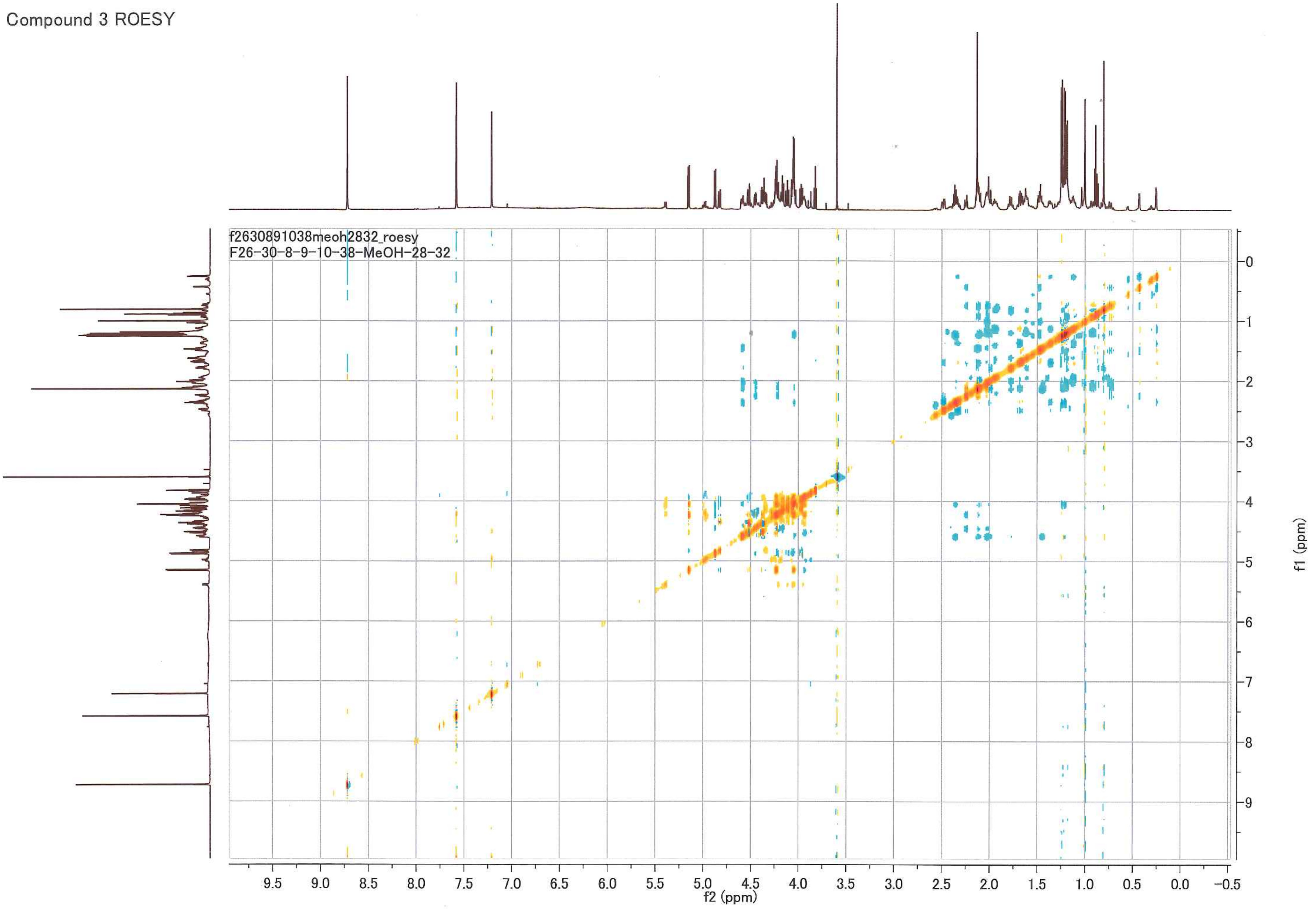

Compound 4 HMQC

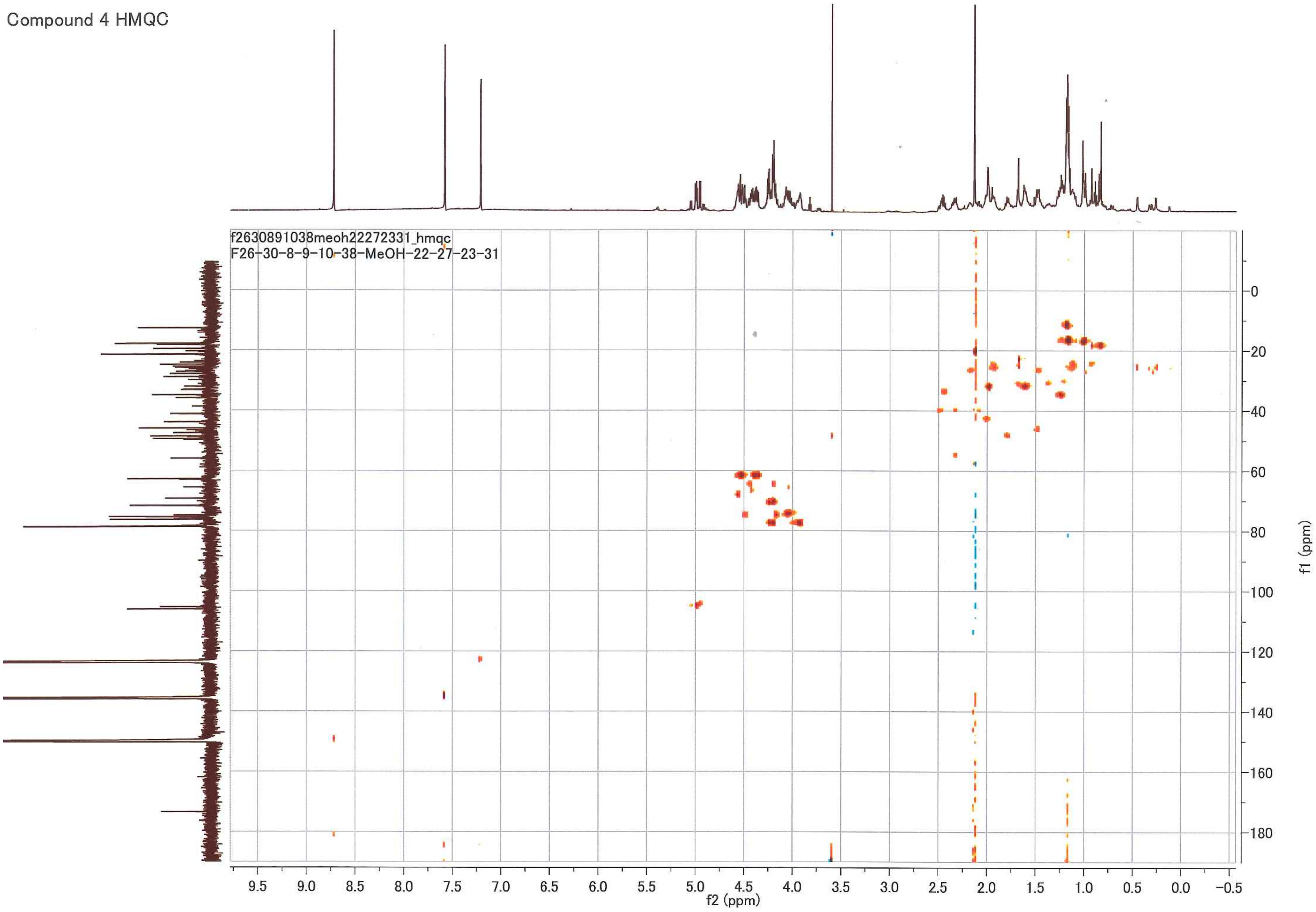

Compound 4 COSY

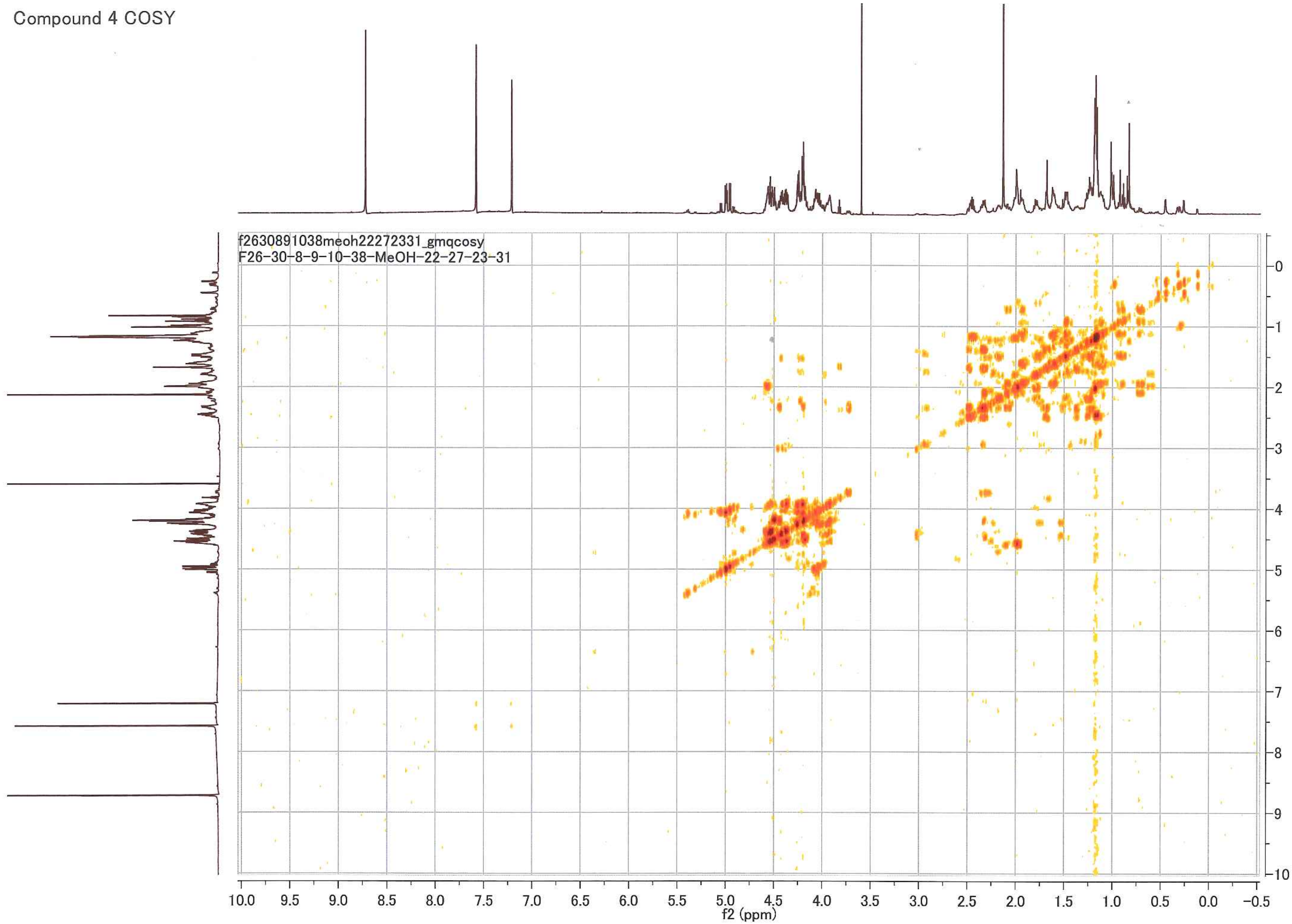

Compound 4 HMBC

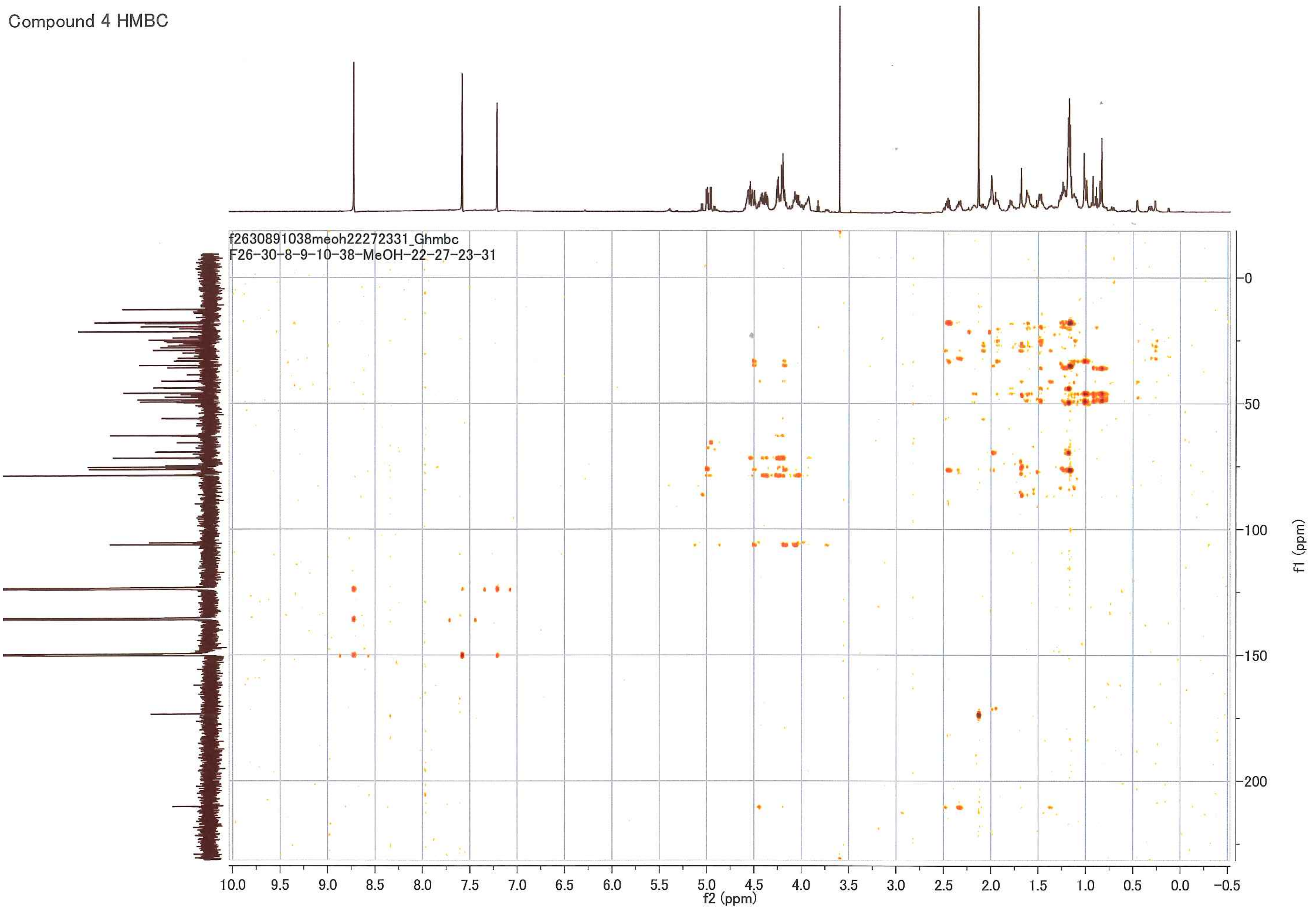

Compound 4 ROESY

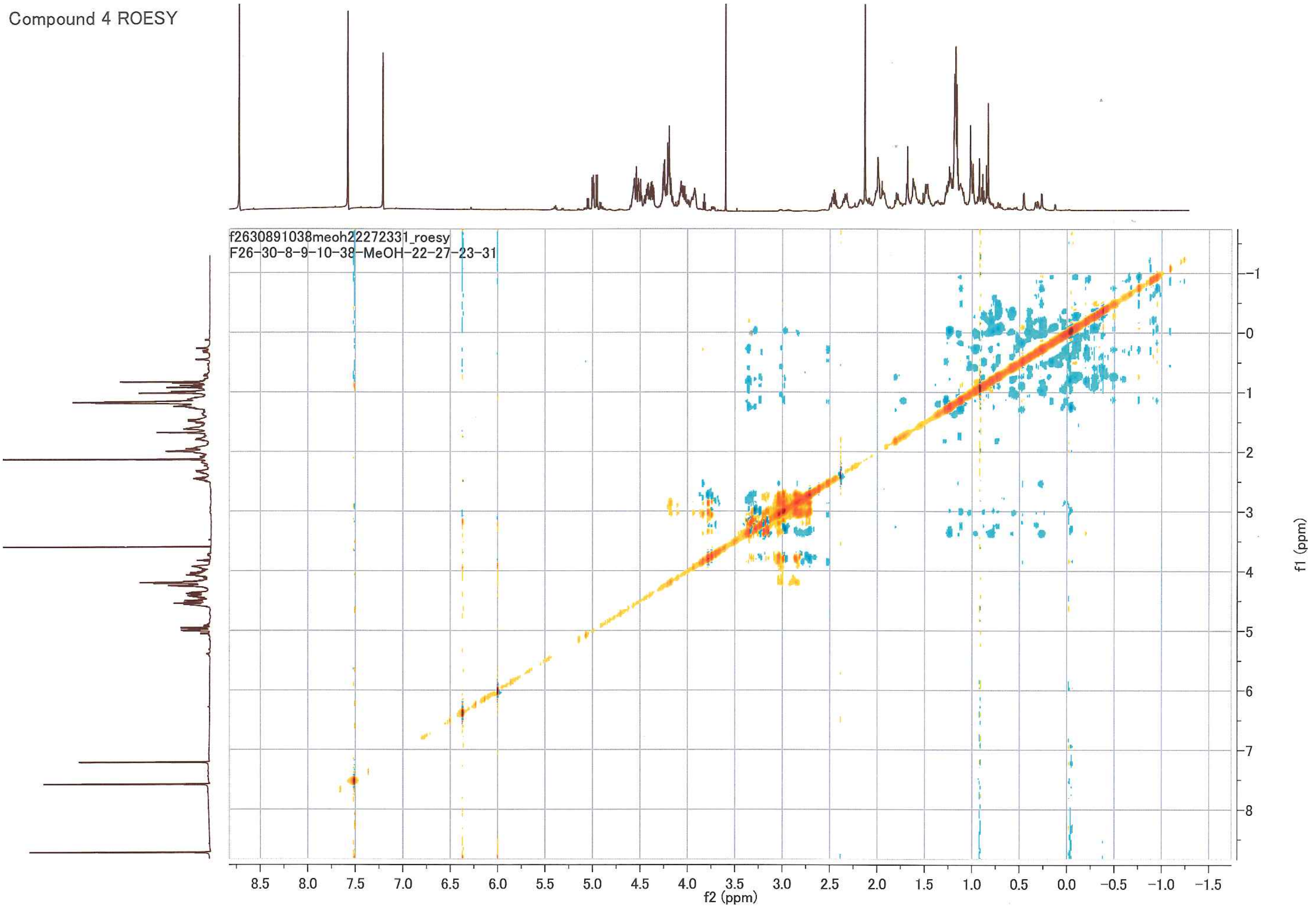

Compound 5 HMQC

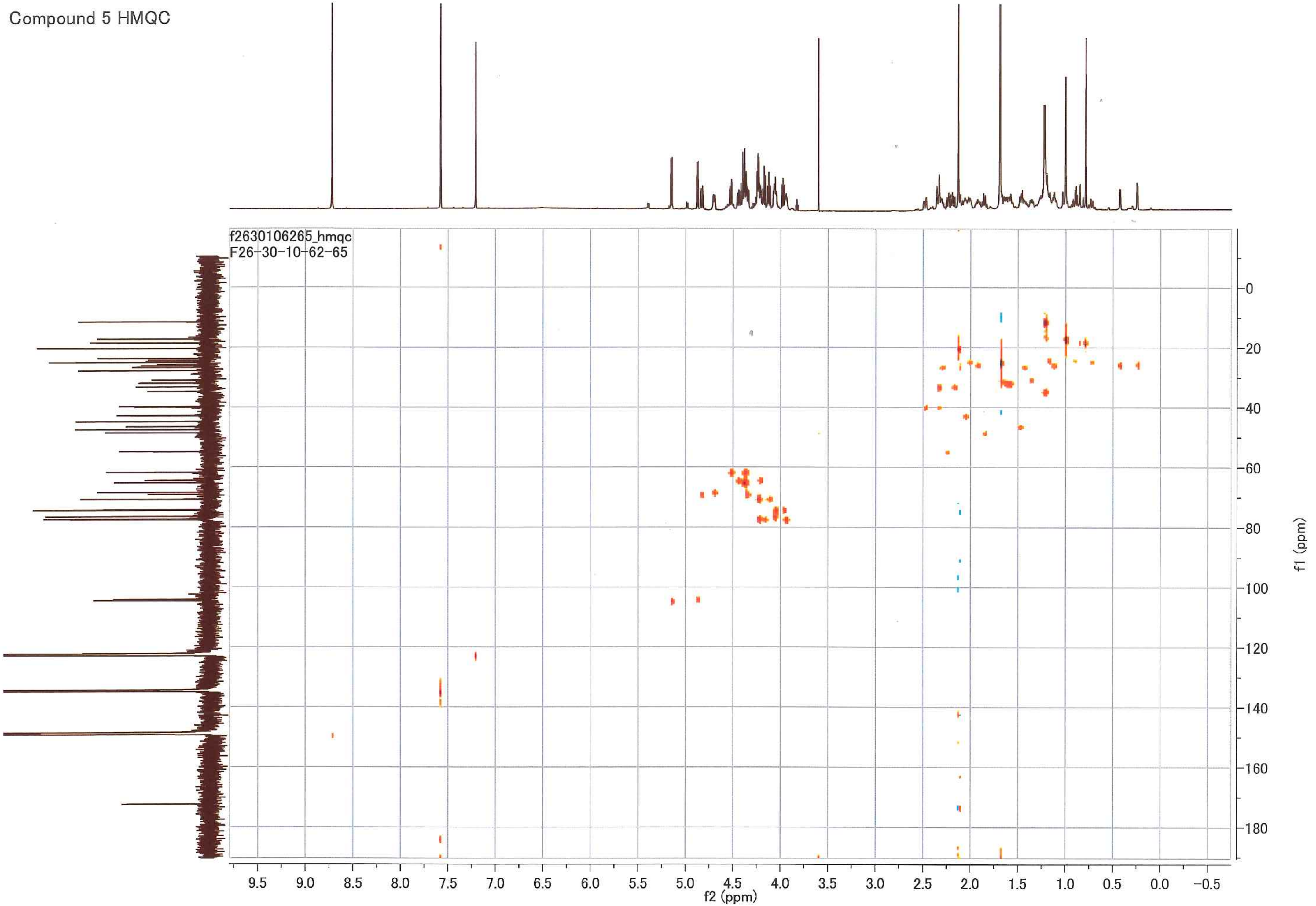

Compound 5 COSY

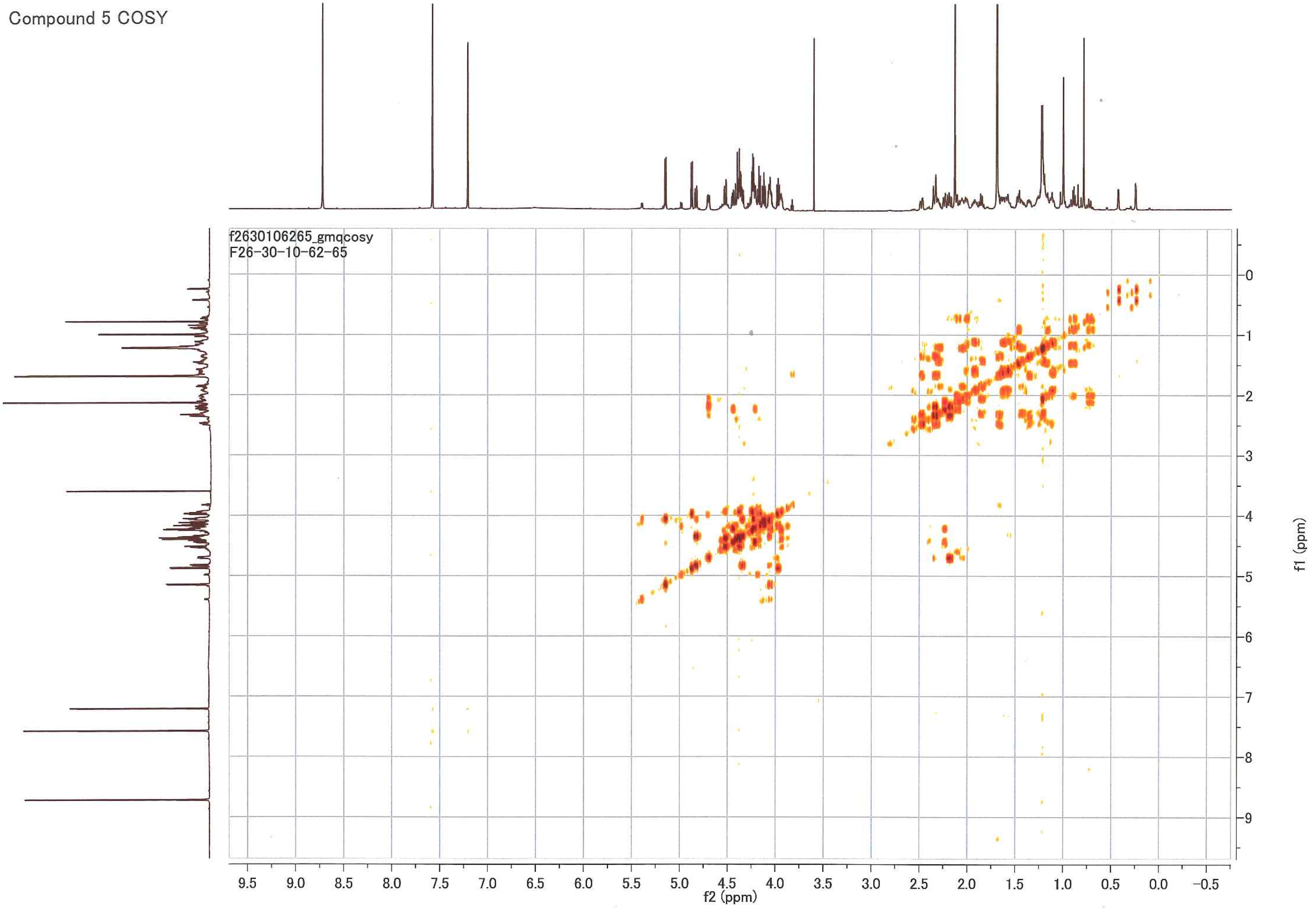

Compound 5 HMBC

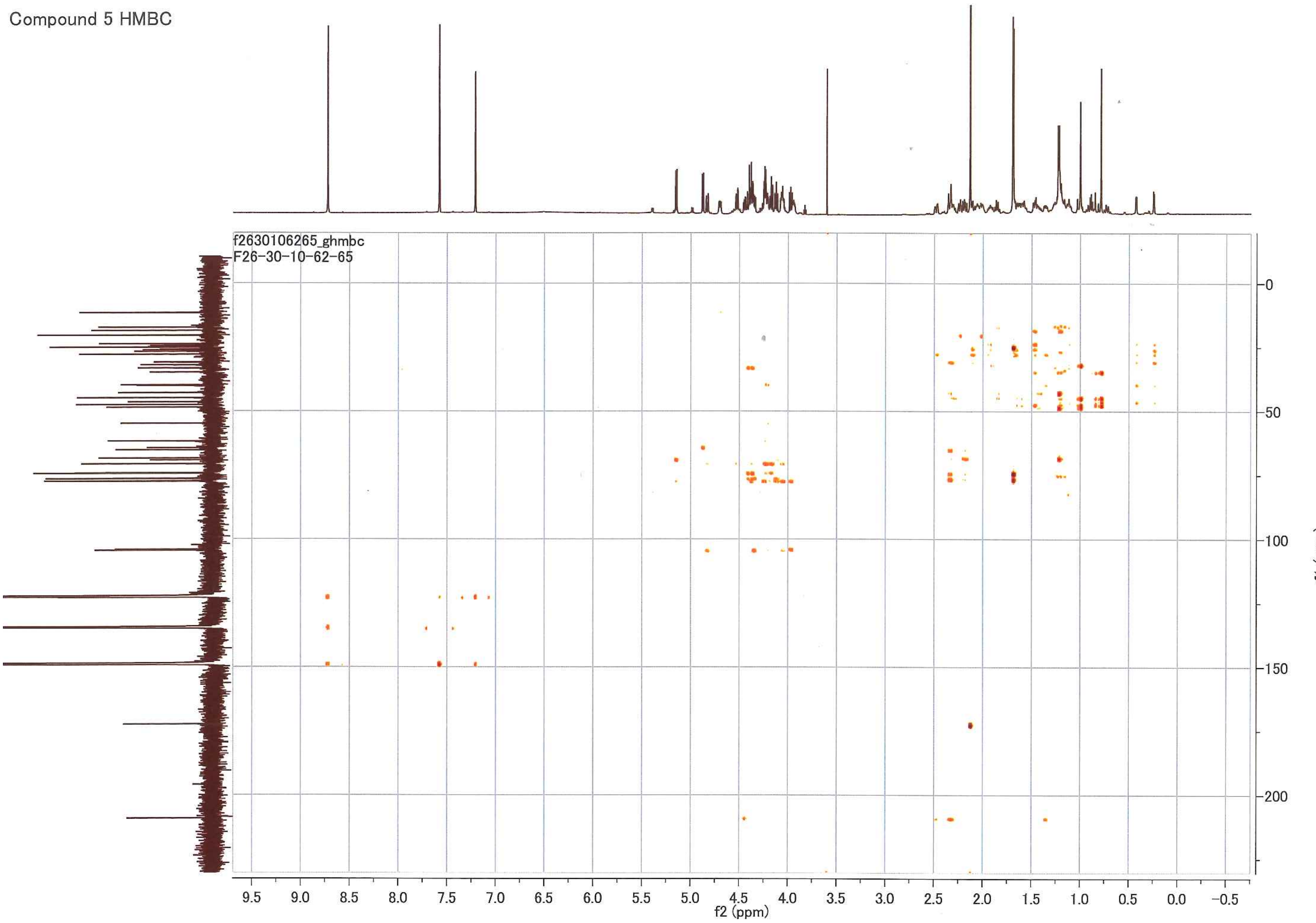

Compound 5 ROESY

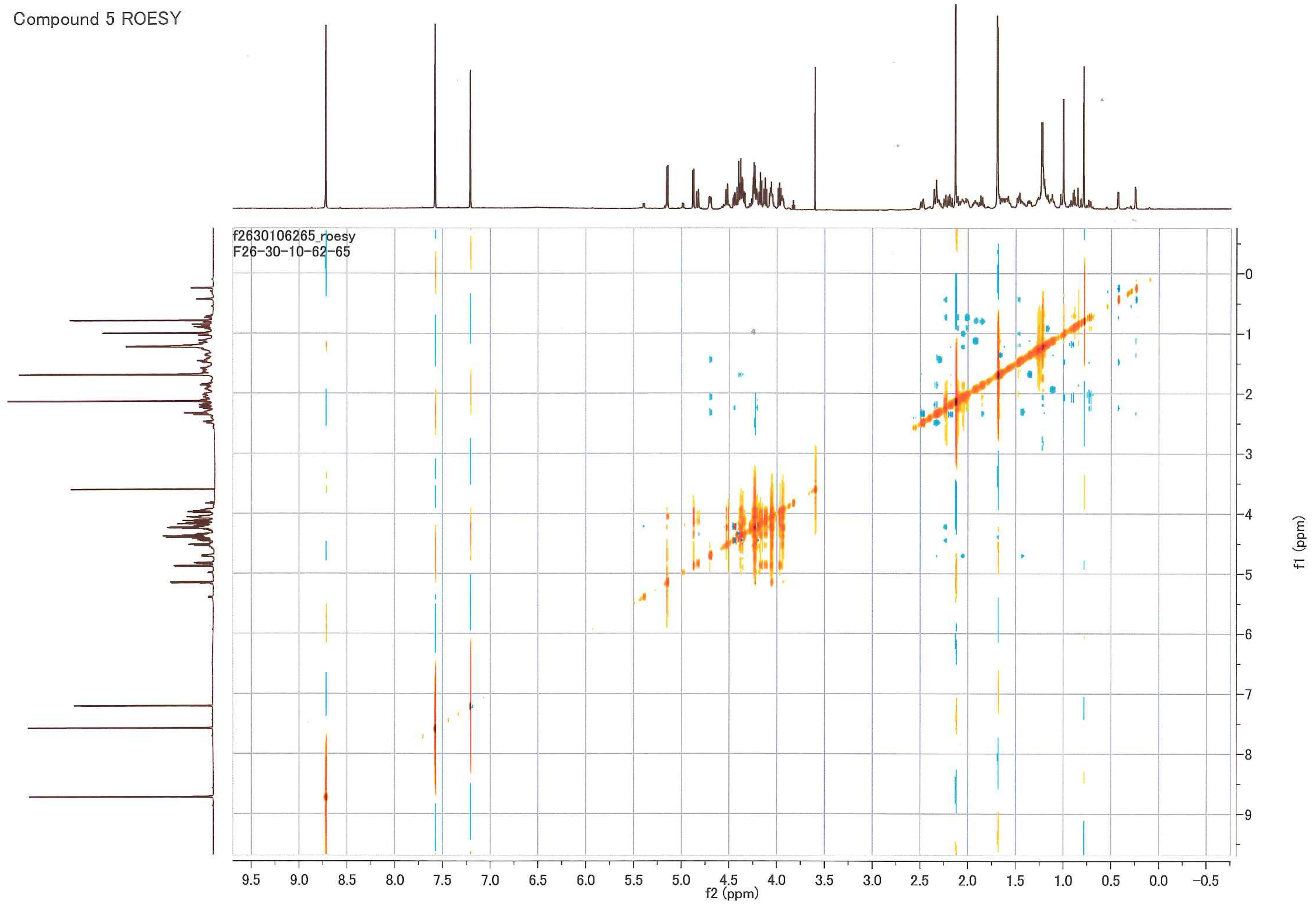

Compound 6 HMQC

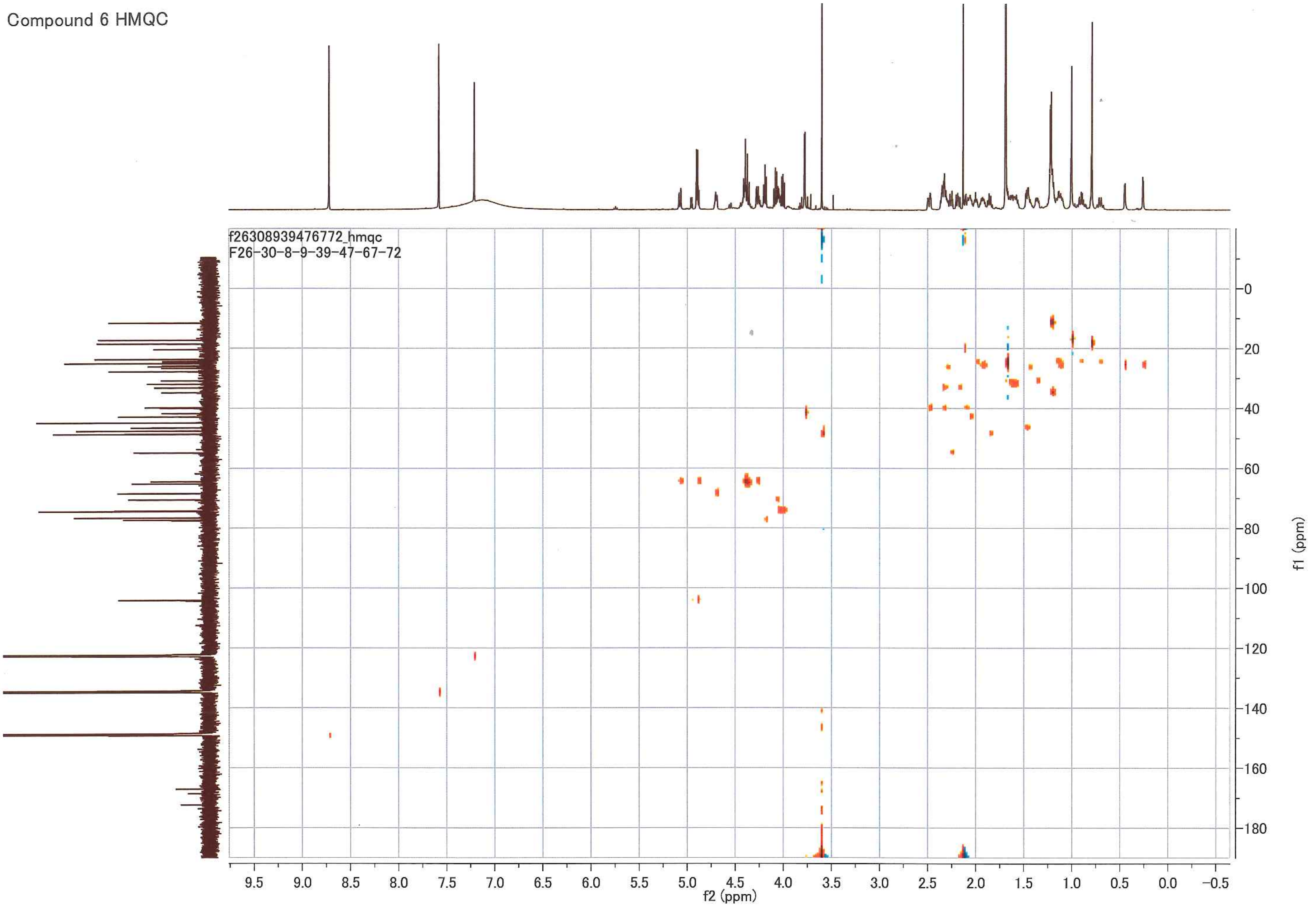

Compound 6 COSY

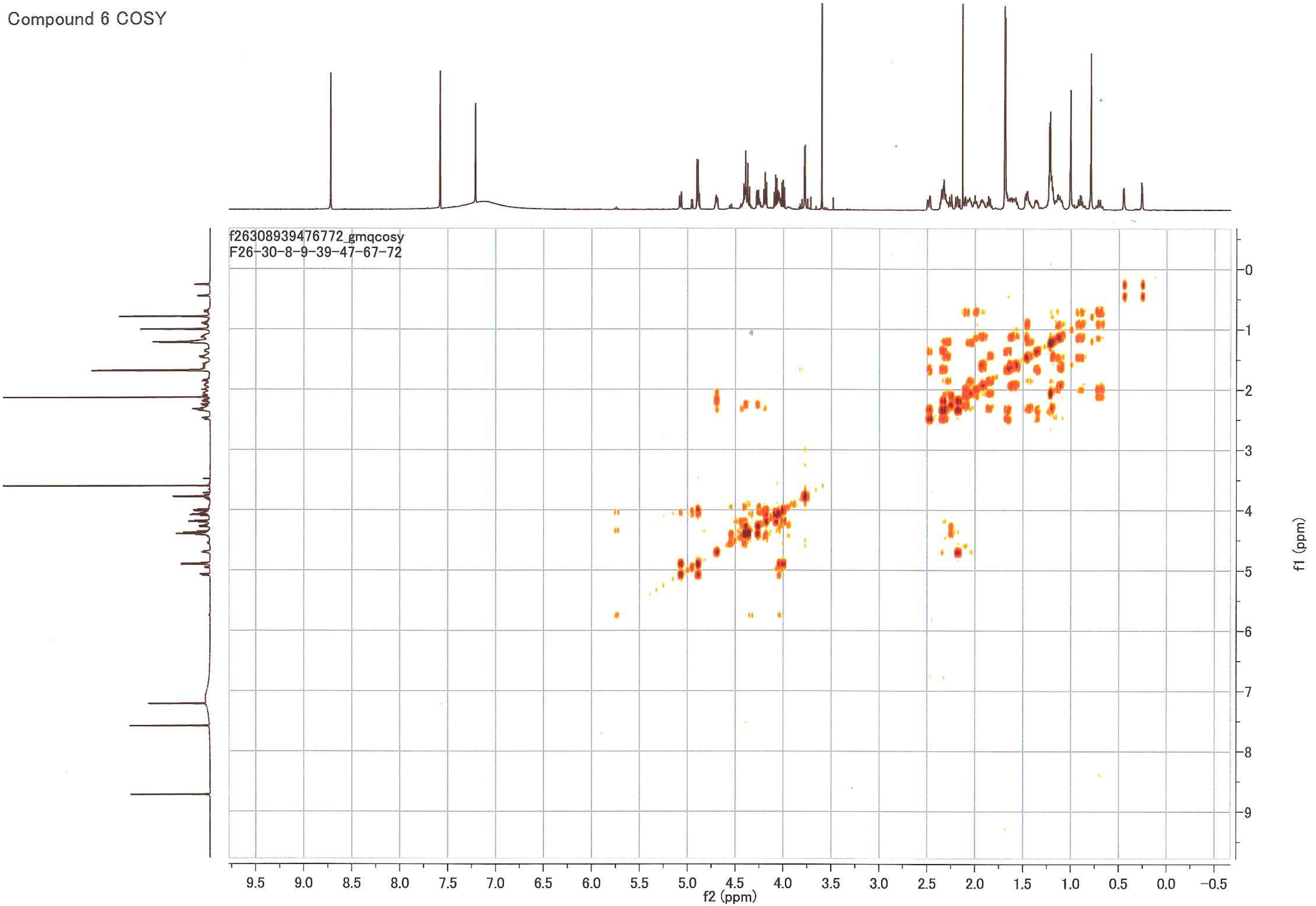

Compound 6 HMBC

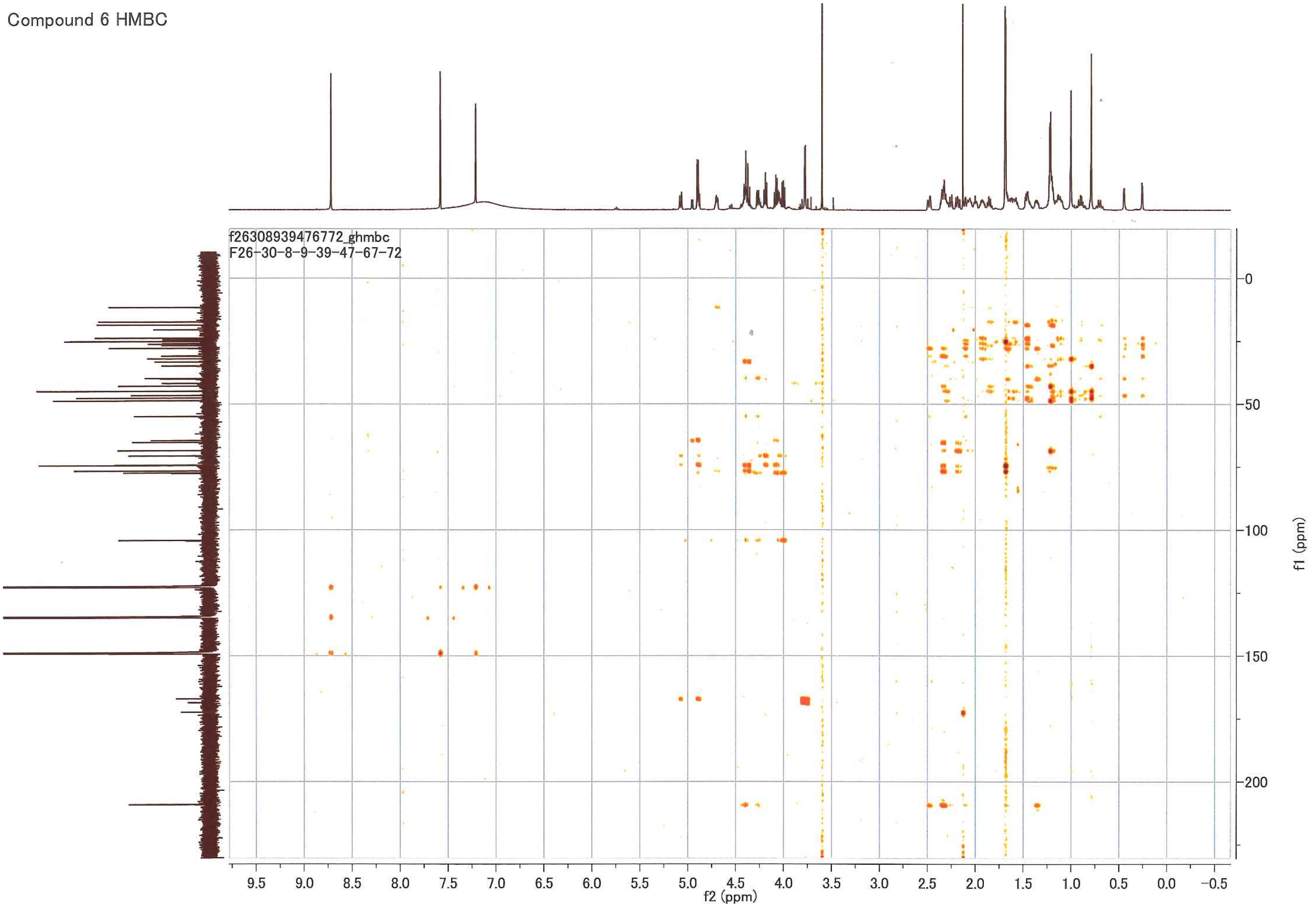

Compound 6 ROESY

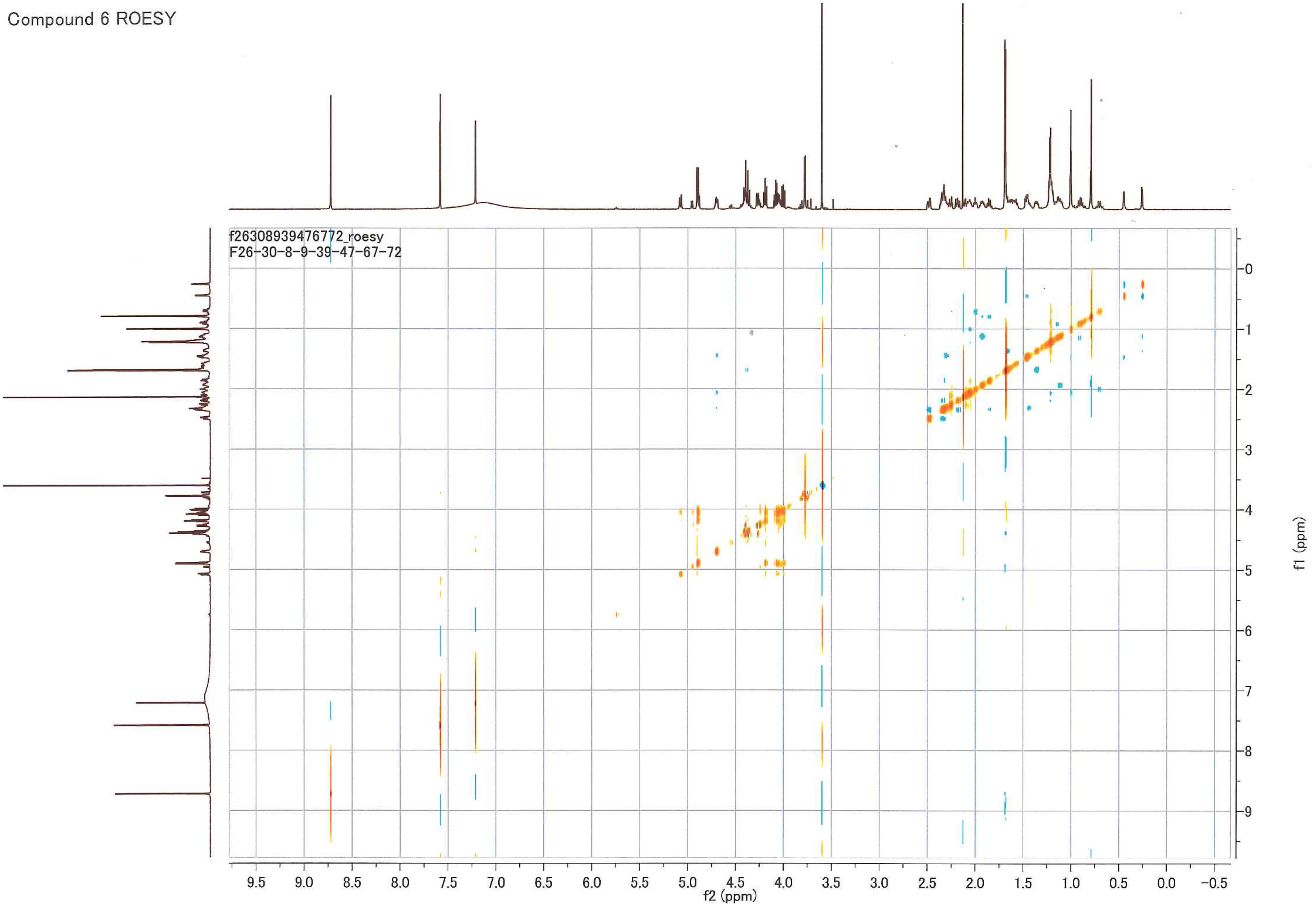

Compound 7 HMQC

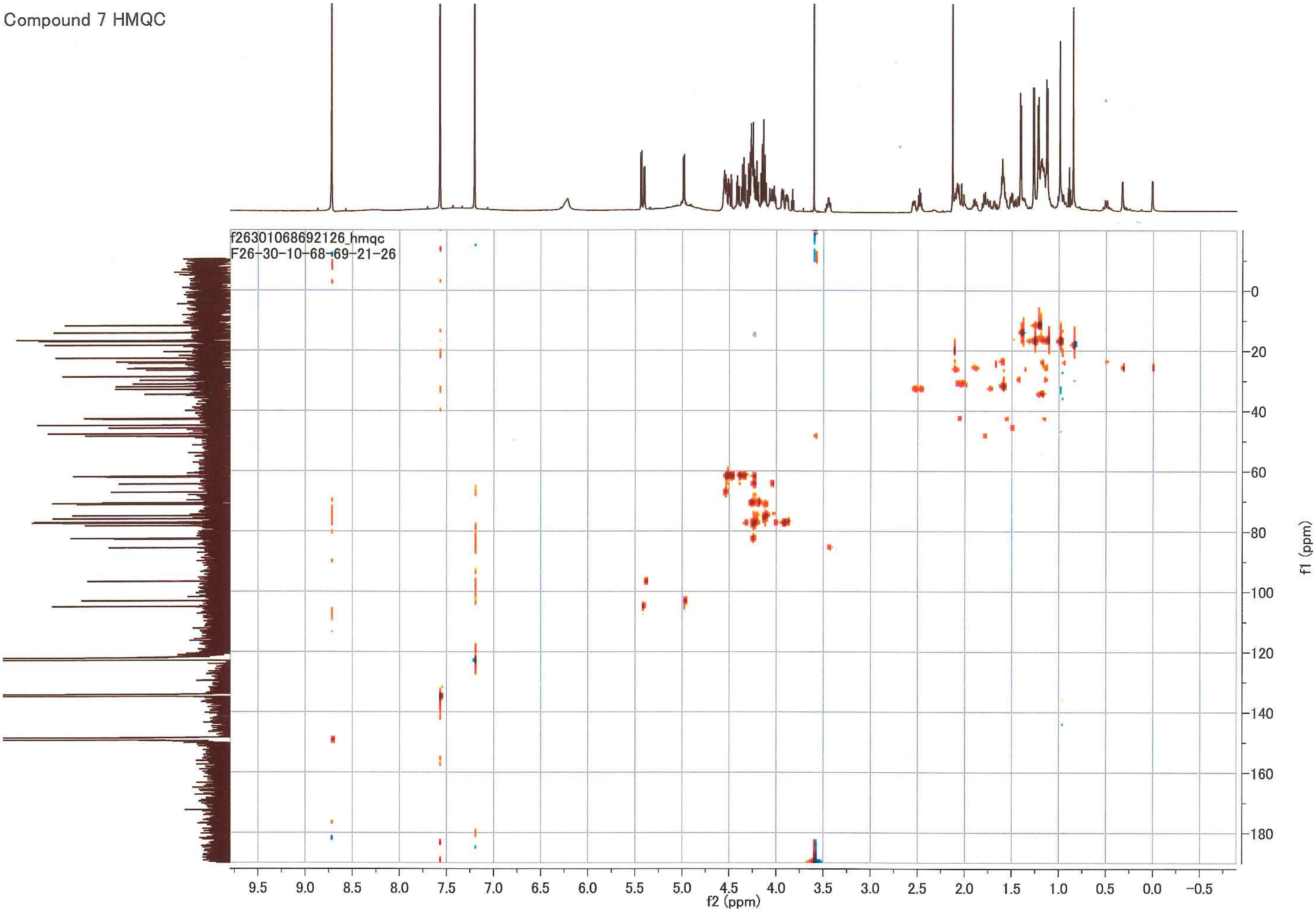

Compound 7 COSY

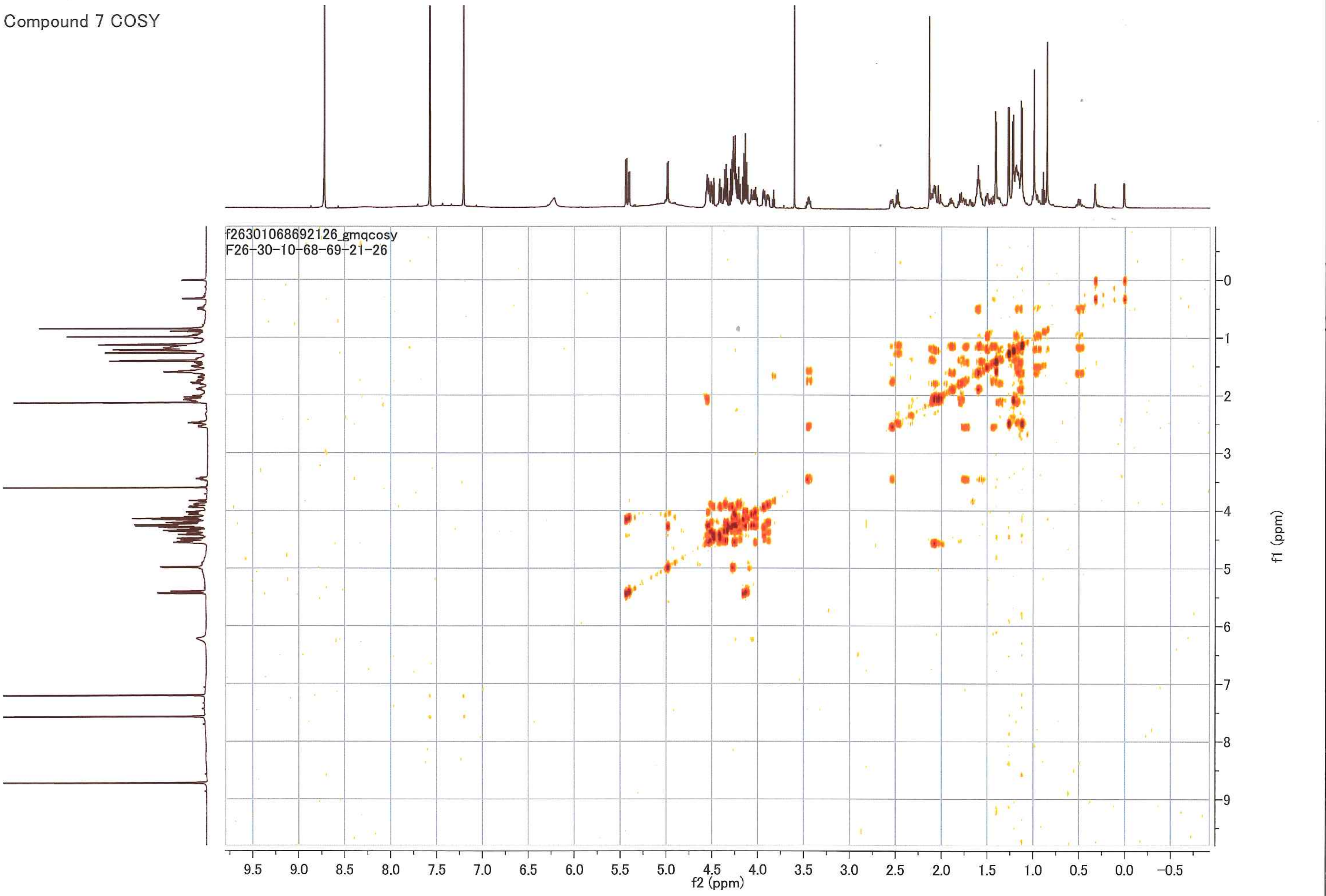

Compound 7 HMBC

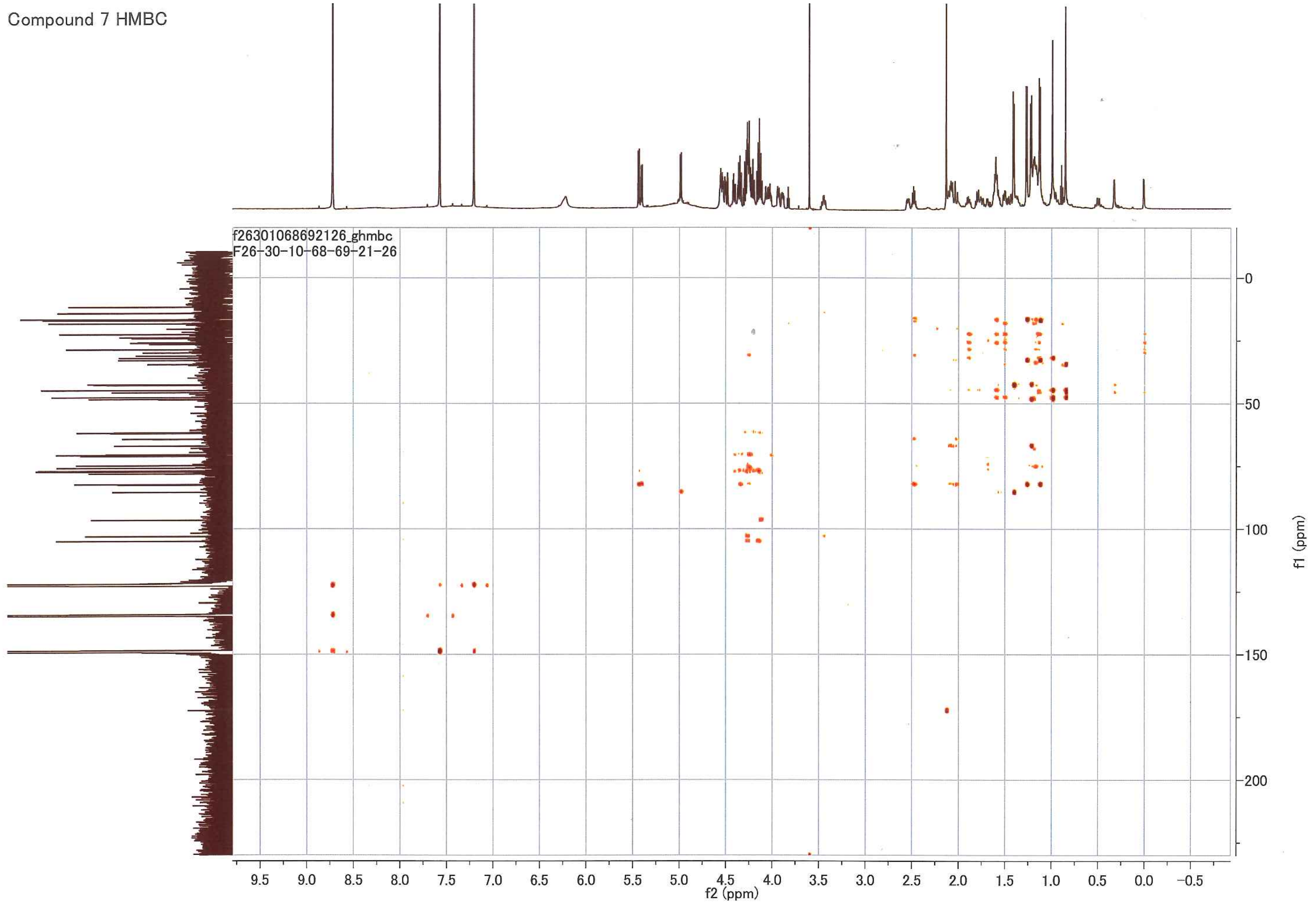

Compound 7 ROESY

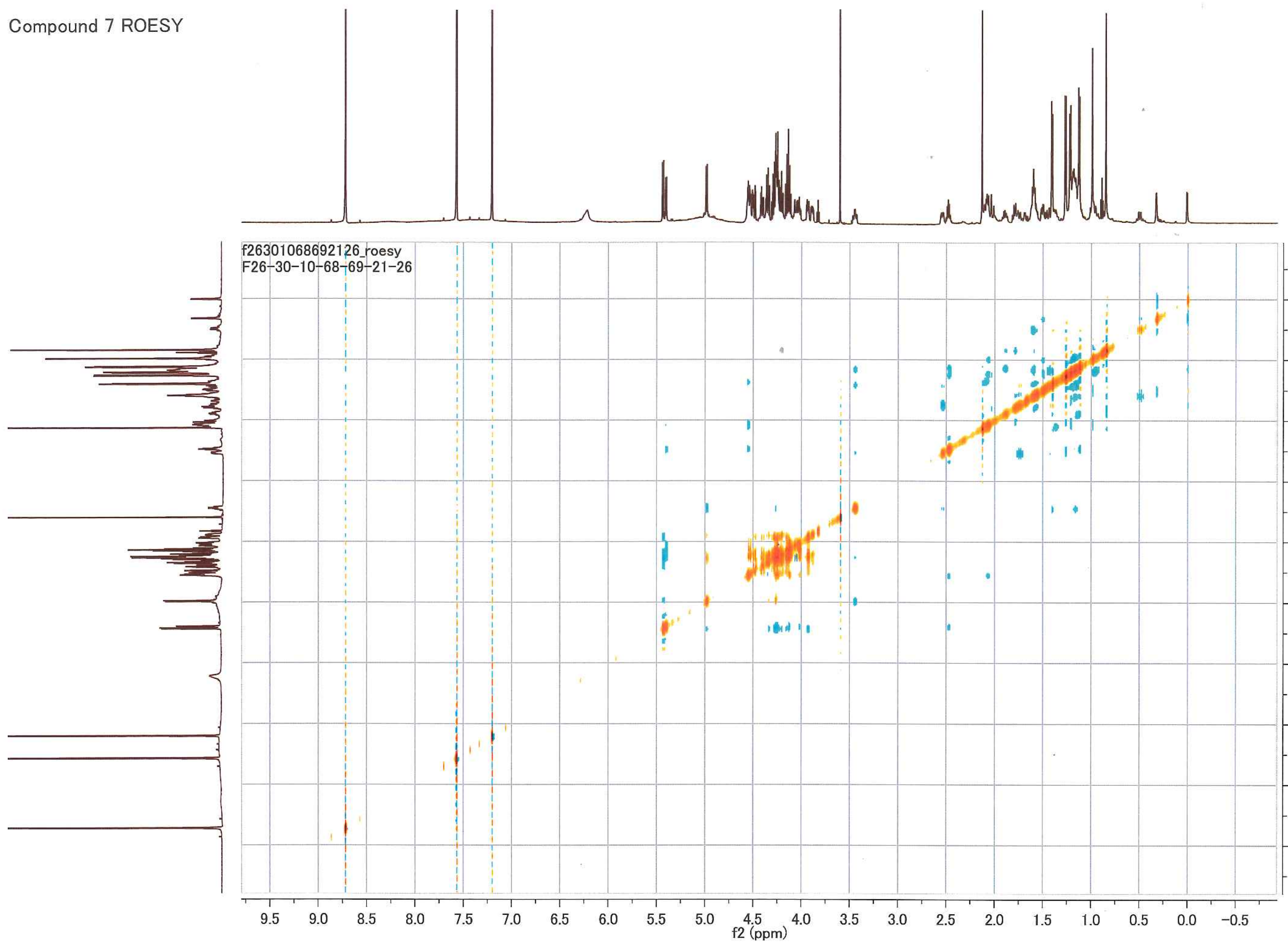

Supplement: Supplementary file 1 [file molecules-24-02504-s001.pdf]
